# Glycoproteomics Landscape of Asymptomatic and Symptomatic Human Alzheimer’s Disease Brain

**DOI:** 10.1016/j.mcpro.2022.100433

**Published:** 2022-10-27

**Authors:** Suttipong Suttapitugsakul, Kathrin Stavenhagen, Sofia Donskaya, David A. Bennett, Robert G. Mealer, Nicholas T. Seyfried, Richard D. Cummings

**Affiliations:** 1 Department of Surgery, Beth Israel Deaconess Medical Center, Harvard Medical School, Boston, Massachusetts, USA; 2 Rush Alzheimer's Disease Center, Rush University Medical Center, Chicago, Illinois, USA; 3 Psychiatric and Neurodevelopmental Genetics Unit, Department of Psychiatry, Massachusetts General Hospital, Harvard Medical School, Boston, Massachusetts, USA; 4 Department of Biochemistry, Emory University School of Medicine, Atlanta, Georgia, USA

**Keywords:** glycoproteomics, Alzheimer’s disease, symptomatic, asymptomatic, lectin enrichment, N-linked glycosylation, AD, Alzheimer's disease, APP, amyloid precursor protein, Aβ, β-amyloid, ConA, Concanavalin A, FDR, false discovery rate, HILIC, hydrophilic interaction chromatography, RCA, *Ricinus communis* agglutinin I, SNA, *Sambucus nigra* agglutinin, SPE, solid-phase extraction, TBS, Tris-buffered saline, WGA, wheat germ agglutinin

## Abstract

Molecular changes in the brain of individuals afflicted with Alzheimer's disease (AD) are an intense area of study. Little is known about the role of protein abundance and posttranslational modifications in AD progression and treatment, in particular large-scale intact N-linked glycoproteomics analysis. To elucidate the N-glycoproteome landscape, we developed an approach based on multi-lectin affinity enrichment, hydrophilic interaction chromatography, and LC-MS–based glycoproteomics. We analyzed brain tissue from 10 persons with no cognitive impairment or AD, 10 with asymptomatic AD, and 10 with symptomatic AD, detecting over 300 glycoproteins and 1900 glycoforms across the samples. The majority of glycoproteins have N-glycans that are high-mannosidic or complex chains that are fucosylated and bisected. The Man5 N-glycan was found to occur most frequently at >20% of the total glycoforms. Unlike the glycoproteomes of other tissues, sialylation is a minor feature of the brain N-glycoproteome, occurring at <9% among the glycoforms. We observed AD-associated differences in the number of antennae, frequency of fucosylation, bisection, and other monosaccharides at individual glycosylation sites among samples from our three groups. Further analysis revealed glycosylation differences in subcellular compartments across disease stage, including glycoproteins in the lysosome frequently modified with paucimannosidic glycans. These results illustrate the N-glycoproteomics landscape across the spectrum of AD clinical and pathologic severity and will facilitate a deeper understanding of progression and treatment development.

## Highlights

• Glycoproteomics analysis of human brains over stages of Alzheimer’s disease.
• Enrichment of N-glycoproteins by multi-lectins and HILIC purification.
• High-mannose and complex glycans that are fucosylated and bisected are predominant.
• Site-specific variation of protein glycosylation among different AD stages.

## Graphical Abstract

**Figure:**
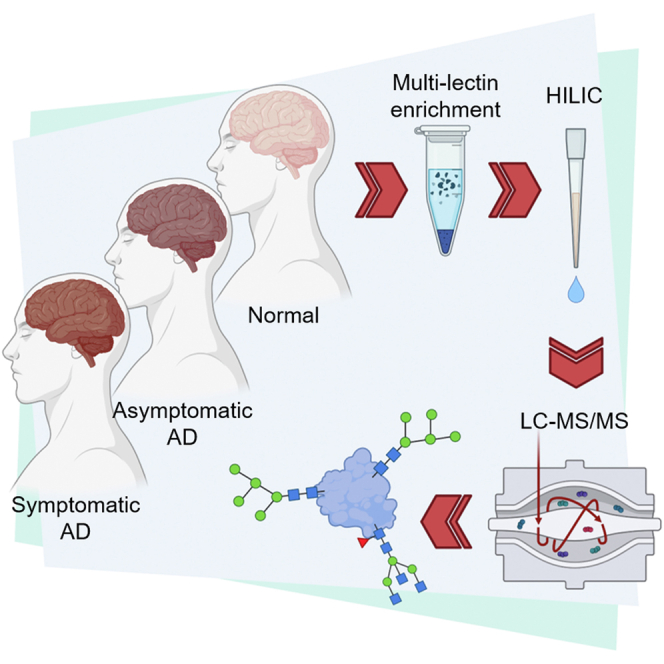

## In Brief

Glycosylation of proteins in human brains, especially those with Alzheimer’s disease (AD) is not well-known. We employed multi-lectin enrichment, HILIC, and mass spectrometry–based proteomics to profile N-glycoproteins in normal, asymptomatic, and symptomatic AD brains. Unlike other organs, the brain consists of mostly high-mannosidic glycans, notably the Man5 N-glycans and complex N-glycans that are fucosylated and bisected. We observed site-specific differences in glycosylation among different AD stages, such as the number of antennae or the frequency of fucosylation.

Alzheimer’s disease (AD) is one of the most common age-related neurodegenerative disorders that affect memory and other mental functions. AD is the most common form of dementia where patients exhibit cognitive decline, short-term memory difficulty, language impairment, and reduced mental agility (1, 2). In the United States, the occurrence of AD is expected to increase from about six million people in 2020 to about 14 million by 2060 (3). The etiology of AD is multifactorial and not well understood. Age is a major risk factor for AD (4). AD is much more common in women, largely due to their longer life expectancy (5, 6, 7). Genetic variation is also linked with the occurrence of AD, in particular, increased risk in carriers of the *APOE* ε4 allele (8, 9). Other metabolic factors, such as diabetes, hypertension, and obesity, have also been correlated with AD, but the connection is still under examination (10, 11, 12). The neuropathological hallmark of AD is the formation of β-amyloid (Aβ)-containing extracellular deposits and intraneuronal neurofibrillary tangles from hyperphosphorylated tau. Aβ is derived from amyloid precursor protein (APP), which is cleaved by beta (BACE1) and gamma secretases (presenilin-1 and 2, nicastrin) into several fragments including Aβ (13, 14). Tau is a microtubule-associated protein that binds to and stabilizes the cytoskeleton. In AD, tau becomes hyperphosphorylated and aggregated into paired helical filaments and eventually neurofibrillary tangles (15). During the asymptomatic stage, accumulation of the plaques and tangles occurs but patients show no cognitive decline (16).

Glycosylation, the enzymatic attachment of carbohydrates to lipids and proteins, has been implicated in AD and its progression. Glycoproteins, glycans, and glycosylation site occupancy from different biological sources including brain, blood, and cerebrospinal fluid (CSF) have been investigated in efforts to reveal mechanisms underlying AD etiology and as potential biomarkers for early diagnostic biomarkers (17, 18, 19, 20, 21). For instance, APP has two potential N-glycosylation sites, and treatment of cells with tunicamycin to inhibit glycosylation or mutation of the two sites affected secretion of soluble APP, suggesting that glycosylation may modulate intracellular sorting of APP (22). Several O-glycosylation sites are also in the proximity of APP’s cleavage site, and modification by polypeptide N-acetylgalactosaminyltransferases has been reported to affect Aβ production (23). Tau is extensively O-GlcNAcylated (24), which regulates its phosphorylation in a site-specific manner, and a decreased O-GlcNAcylation in the brain correlated with tau hyperphosphorylation (25). Interestingly, some proteins such as BACE1, which cleaves APP into Aβ, can also process sialyltransferases, such as ST6GAL1, to initiate its secretion and affect protein sialylation (26). Other studies have employed large-scale approaches to study protein glycosylation in AD, including one investigation of the proteome and glycoproteome of AD and age-matched control brains by hydrophilic interaction chromatography (HILIC) enrichment. Label-free quantification was performed for both the proteome and glycoproteome, allowing for the determination of 36 glycosylation sites on 28 glycoproteins with increased N-glycosylation site occupancy and 41 glycosylation sites on 34 glycoproteins with decreased N-glycosylation site occupancy in AD compared with normal brains (20). However, as PNGase F was used, site-specific glycan information for each glycan was lost. Another work investigated glycoproteins with intact glycans from CSFs of AD patients and age-matched controls (pooled into one sample) by HILIC and boronic acid enrichment. Altered protein glycosylation, particularly a decrease in fucosylation, was found in AD samples. Interestingly, about one-third of the glycopeptides have the same glycosylation pattern (27). Despite these extensive efforts, a more expanded view of protein glycosylation in AD brains, especially with intact glycoprotein/glycopeptide analysis, is lacking.

To investigate the larger glycoprotein landscape, we performed a qualitative N-glycoproteomics analysis of brain samples from 30 humans including those with no cognitive impairment and without pathologic AD, asymptomatic, and symptomatic AD. A combination of lectin affinity isolation and HILIC was optimized and employed to selectively enrich N-glycoproteins prior to mass spectrometry–based intact glycoproteomics analysis. We found that the glycoproteins identified in the brain are mainly high-mannosidic, fucosylated, and contain a bisecting GlcNAc residue. Sialylation, in contrast to other samples such as serum, was not commonly identified in the brain. While the overall glycosylation profile was shared among all tissues, the heterogeneity at individual sites varied by clinical AD stages across the three sample groups, including an altered frequency of protein fucosylation, galactosylation, and the number of antennae and glycans with a bisecting GlcNAc residue. This information provides new insights on the role of protein glycosylation in AD onset and progression, with the potential for altered glycoproteins to serve as diagnostic biomarkers or therapeutic targets.

## Experimental Procedures

### Brain Samples

Optimization of glycoprotein enrichment was performed with brains from C57BL/6 mice in accordance with approved Institutional Animal Care and Use Committee protocols (Beth Israel Deaconess Medical Center, Harvard Medical School). Postmortem human brain tissues from the whole frontal cortex matter were obtained from Rush Alzheimer’s Disease Center’s Religious Orders Study or Rush Memory and Aging Project (ROSMAP) (28). Participants enrolled without known dementia and agreed to annual clinical evaluation and brain donation at the time of death. The studies were approved by an Institutional Review Board of Rush University Medical Center. All participants signed an informed consent, an Anatomic Gift Act, and a repository consent allowing their resources to be shared. At the time of death, a neurologist reviewed all clinical data and rendered a diagnosis of dementia, mild cognitive impairment, referring to persons with cognitive impairment without dementia, and no cognitive impairment, referring to the group without dementia or mild cognitive impairment. Neuropathological evaluations for Braak and CERAD were combined for an NIA-Reagan diagnosis of pathologic AD. Brain samples were obtained for 10 with no cognitive impairment and no pathologic AD and 10 each with and without cognitive impairment who had pathologic AD. This included brains from both males and females. Information on the patient’s age, sex, Braak stage, CERAD score, race, diagnosis, and others can be found in supplemental Table S1.

### Sample Preparation and Protein Extraction

Reagents and materials were obtained from Sigma unless noted otherwise. The brain tissues were lysed in 2 ml Tris-buffered saline (TBS) containing 0.5% Triton X-100 and cOmplete, mini EDTA-free protease inhibitor cocktail (Roche) using a Brinkmann homogenizer twice at level 5 on ice, 20 s each. The samples were then sonicated three times with a Qsonica sonicator for 10 s each at 30 Amps with 30 s break between rounds. The lysates were centrifuged at 2000*g* for 20 min at 4 °C. The supernatants were collected. Bicinchoninic acid (BCA) assay was performed to determine the protein concentration. The lysates were used immediately or stored at −80 °C until use.

### Multi-Lectin Enrichment of Brain Glycoproteins

A combination of six lectins was used to enrich glycoproteins from the brain lysates by mixing agarose-bound derivatives of specific lectins. These included 250 μl Concanavalin A (ConA), 80 μl *Ricinus communis* agglutinin I (RCA), 80 μl *Sambucus nigra* agglutinin (SNA), 90 μl *Aleuria aurantia* lectin, 120 μl wheat germ agglutinin (WGA), and 80 μl *Vicia villosa* agglutinin (Vector Laboratories). The beads were washed four times with wash buffer containing 1 mM CaCl_2_, 1 mM MnCl_2_, 0.1% Triton X-100, and protease inhibitor cocktail in TBS, and centrifuged at 500*g* for 30 s to remove the liquid. One milligram of brain proteins from each lysate according to the BCA assay was added to the lectin beads and incubated on a rotator at 4 °C overnight. After the enrichment, the beads were washed four times with wash buffer. The enriched glycoproteins were eluted with 1) TBS containing 200 mM methyl α-D-mannopyranoside, 200 mM methyl α-D-glucopyranoside, 200 mM D-(+)-galactose, 100 mM L-(-)-fucose, 500 mM *N*-acetyl-D-glucosamine, 200 mM *N*-acetyl-D-galactosamine (Carbosynth), 0.1% Triton X-100, and protease inhibitor, 2) TBS containing 500 mM D-lactose (Fisher), 0.1% Triton X-100, and protease inhibitor, and 3) TBS containing 500 mM D-lactose and 200 mM acetic acid (Chem-Impex). Each elution was incubated for 15 min with shaking at 4 °C. The last eluate was pooled and neutralized immediately with 1 M Tris–HCl, pH = 8.7. The wash solutions and pooled eluates were run on SDS-PAGE to confirm that the wash and elution steps were efficient.

### Sample Cleanup and Tryptic Digestion with Filter-Aided Sample Preparation

Filter-aided sample preparation was performed to remove the detergent and digest the proteins as reported previously (29). Briefly, the eluates were transferred to a 30 kDa Microcon centrifugal filter unit (Millipore) and centrifuged at 14,000*g*, 4 °C to remove the liquid. The buffer was exchanged to the one containing 8 M urea in TBS. The proteins were reduced with 10 mM DTT at 37 °C for 1 h in 8 M urea in TBS and subsequently alkylated with 50 mM iodoacetamide for 20 min at room temperature in the dark. The proteins were washed with 25 mM ammonium bicarbonate solution four times. Eventually, the volume was adjusted to 100 μl, and the proteins were digested with sequencing-grade modified trypsin (Promega) at 37 °C with shaking overnight. The peptides were spun down, collected, and lyophilized. Ten percent of the peptides were analyzed with LC-MS/MS to generate a focused proteome database for glycoproteomics analysis.

### HILIC SPE Enrichment of Glycopeptides

After protein digestion, HILIC solid-phase extraction (SPE) was employed to enrich glycopeptides before LC-MS/MS analysis. Cellulose microcrystalline slurry in 50% acetonitrile (ACN) was packed into an empty TT1 TopTip (Glygen) until the bed volume is approximately 1.5 cm high. The material was washed with 0.1% TFA and equilibrated with 0.1% TFA in 80% ACN. The dried glycopeptides were dissolved in water, brought to the equilibration solution, and loaded into the tip. The flow through was also reloaded into the column twice to ensure that all glycopeptides were enriched. The microcrystalline was washed with the equilibration solution, and the glycopeptides were eluted with 0.1% TFA. The wash, equilibration, and elution steps were performed three times each. The eluted peptides were dried and lyophilized before LC-MS/MS analysis.

### LC-MS/MS Analysis

The dried peptides were dissolved in 0.1% formic acid (FA) and analyzed with LC-MS/MS as described elsewhere with slight adjustments (30). The peptides were loaded onto a C18 PepMap 100 (300 μm × 5 mm, 5 μm, 100 Å precolumn, Thermo) using 15 μl/min 0.1% FA for 3 min and separated with a PicoFrit analytical column (75 μm ID × 150 mm, 3 μm, New Objective) using a gradient of 2% to 37.2% of 80% ACN with 0.1% FA over 92 min and 37.2% to 90% over 3 min at 400 nl/min on a Dionex UltiMate 3000 UHPLC system (Thermo). The peptides were analyzed with an Orbitrap Fusion Lumos Tribrid mass spectrometer (Thermo) in a positive mode. The spray voltage was set to 2100 V and the transfer tube temperature was 200 °C. For each cycle, a full MS spectrum was recorded at the resolution of 60,000 in the Orbitrap with the scan range of *m/z* 400 to 1,600, RF lens of 30%, AGC target of 1e5, and a maximum ion injection time of 50 ms. Data-dependent acquisition was performed with the top 15 most intense ions selected for MS (2) analysis. For proteomics experiments, higher energy collision dissociation (HCD) was used to fragment the peptides with 28% normalized collision energy. The isolation window was set to 1.2 *m/z*. For glycoproteomics experiments, the peptides were fragmented with stepped HCD with 30 ± 5% normalized collision energy. The isolation window was set to 1.5 *m/z*. The fragments were recorded in the Orbitrap with a resolution of 30,000. For the optimization experiments, a gradient from 2% to 45% of 80% ACN with 0.1% FA over 106 min and 45% to 90% over 3 min at 400 nl/min was used instead. Other MS parameters were the same.

### Database Searching, Data Filtering, and Bioinformatics Analysis

For the proteomics experiment, the raw files were searched against the Swiss-Prot human proteome database downloaded from UniProt (June 18, 2021, reviewed, 42,387 entries), which contains both canonical and noncanonical protein sequences. Byonic (version 4.1.10, Protein Metrics) was used to search for the proteins present in each sample after multi-lectin enrichment and tryptic digestion. Trypsin was set as an enzyme with fully tryptic peptides and two maximum missed cleavages required. The precursor mass tolerance was 10 ppm and the fragment mass tolerance was 0.025 Da. Carbamidomethylation of cysteine (+57.0215 Da) was set as a fixed modification while oxidation of methionine (+15.9949 Da) was set as a common variable modification (common2). Results were filtered at a 1% protein false discovery rate (FDR). The proteins were combined from all runs, reverse hits and contaminants were removed, and the final protein list was used to generate a focused FASTA database for the glycoproteomics experiments. In the glycoproteomics experiment, similar parameters were employed for the search of intact glycopeptides after HILIC enrichment. However, the focused FASTA database from the proteomics experiments was employed for the search. N-linked glycosylation was searched using the “N-glycan 309 mammalian no sodium” database from Byonic. Results were filtered at a 1% FDR. Due to the FDR control of Byonic for intact glycopeptide identification, the glycopeptides were further filtered and manually inspected so that they have a 1D PEP score ≤ 0.001, |log prob|>4, score >300, and ppm <10. Raw files from the initial optimization experiments were also searched similarly, except that the UniProt proteome database from mouse (*Mus musculus*, June 18, 2021, reviewed, 25,368 entries) was used directly without the focused proteome database generated. Statistical analysis was performed with Origin Pro 2021. Functional analysis and tissue expression enrichment were performed with FUMA (31). Glycan type was annotated from the composition according to Williams *et al*. (32) or GlyGen (33). Solvent accessibility for asparagine residues was predicted with NetSurfP version 1.1 (34). Sequence motifs were generated with pLogo (35).

### Experimental Design and Statistical Rationale

Due to their low abundance, glycoproteins must be enriched prior to MS analysis. We first optimized the glycoprotein enrichment method with mouse brain (N = 1 for each condition, no replication was performed to compared the results), including the selection of lectins for the enrichment, due to the similarity between mouse and human brains. The approach combining lectin affinity isolation and HILIC SPE enrichment was then applied to study human brain samples, including five normal males, five normal females, five asymptomatic AD males, five asymptomatic AD females, five symptomatic AD males, and five asymptomatic AD females, which should represent an accurate representation of the glycoproteome of the brain. For two brain samples, technical triplicates were performed to evaluate the enrichment approach and reproducibility. Each sample was analyzed once with LC-MS/MS. Statistical tests, including *t* test and ANOVA, were employed when appropriate (see details in the results section) with Benjamini-Hochberg correction of *p*-values reported.

## Results

### Analysis of Glycoproteins from Brain Samples with Multi-Lectin Affinity Enrichment and HILIC

Prior studies on AD brains revealed some alterations of proteins and pathways compared with normal brains (36, 37, 38). Proteins can be extensively posttranslationally modified, for example, through glycosylation, phosphorylation, and ubiquitination, resulting in alterations of their biological functions and downstream signaling networks that may contribute to disease progression (39, 40, 41). Analysis of protein glycosylation in AD, in particular, is often performed with single proteins in model systems, and thus there is a lack of information about the global glycosylation landscape in the brain. Because of their low abundance in biological samples, we reasoned that brain glycoproteins should be enriched to aid in large-scale glycoproteomics analysis. We first tested the enrichment of these glycoproteins from mouse brains by a combination of lectins due to their high glycan specificity and subsequently with HILIC prior to LC-MS analysis. Lectin enrichment for glycopeptides and glycoproteins has a history of success, depending on the combinations of lectins used (42, 43, 44, 45, 46).

In our initial experiments, with a combination of ConA, RCA, SNA, and without the subsequent HILIC separation, we found that several glycosylated peptides especially fucosylated glycopeptides were not effectively captured and remained in the flow through (supplemental Fig. S1 and supplemental Table S2). Nevertheless, the subsequent HILIC separation improved the coverage by removing nonglycosylated peptides from the samples (supplemental Table S2). Eventually, based on our recent studies of the murine brain N- and O-glycome and glycoproteome and the predicted and observed specificities of these lectins (32, 47, 48), we found that a combination of ConA, RCA, SNA, WGA, *V. villosa* agglutinin, and *A. aurantia* lectin could target the major monosaccharide components of N-glycans in the brains. To evaluate the enrichment efficiency of these six lectins, the flow through after the enrichment was subjected to a second round of enrichment. SDS-PAGE and LC-MS analysis of the HILIC SPE-enriched fractions showed that most of the glycoproteins were effectively captured after the first enrichment (supplemental Fig. S2 and supplemental Table S3). The starting protein amount required for effective enrichment was also evaluated (supplemental Fig. S2). Even with a lower starting protein amount, there was always a minor portion of glycoproteins that was not captured by the first enrichment. Additionally, a comparison of different enrichment platforms revealed that the lectin enrichment can simply be done in a centrifuge tube without the need for a spin column or filter devices and yielded reproducible results. We tested this workflow comprising the lectin glycoprotein enrichment and HILIC glycopeptide enrichment using three human brain samples, including one from normal, asymptomatic AD, and symptomatic AD patients (supplemental Fig. S3). The protein amount loaded onto the gel was compared between 0.5 and 1 mg. As expected, the 1 mg load resulted in more intense gel bands. A second enrichment with the flow through was also performed, and the result showed that the enrichment is still effective at a higher protein load with minimal protein background from the second enrichment. Thus, 1 mg was used as the starting amount in the cohort. Glycoproteomics analysis based on stepped HCD fragmentation, which is beneficial for intact N-glycopeptide analysis (49), of the three human brain samples resulted in the identification of >100 glycoproteins from hundreds of glycopeptides with intact glycans (supplemental Fig. S4), showing that our method is effective. Overall, we found this approach to be both facile and successful using the test samples, and thus we applied it to study glycoproteins from the cohorts.

### Identification of Glycoproteins from Normal, Asymptomatic, and Symptomatic AD Brains

**Fig. 1:**
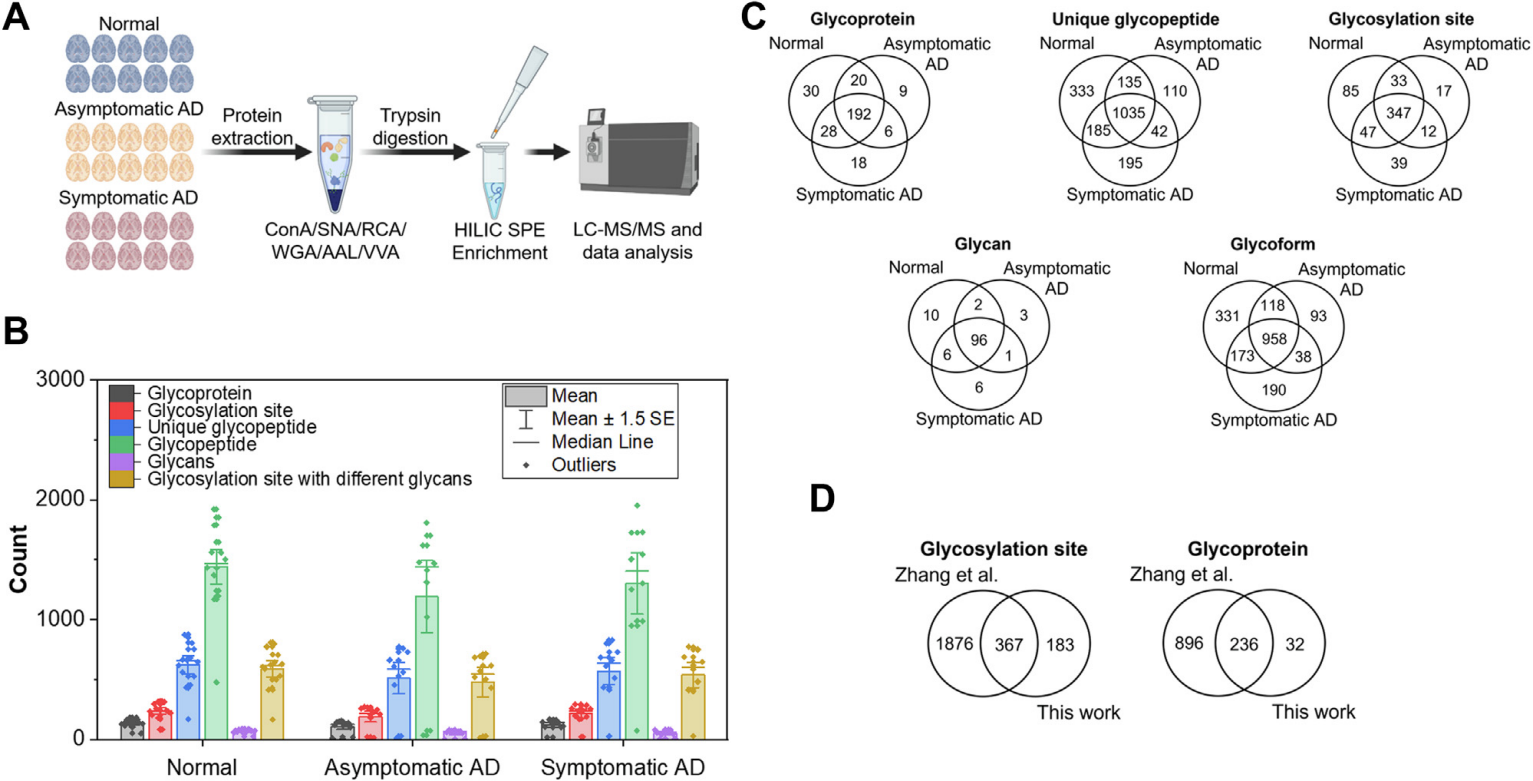
**Identification of N-glycoproteins from human brains.***A*, experimental procedure overview. Proteins from 30 normal, asymptomatic AD, and symptomatic AD human brains were first extracted. Glycoproteins were enriched with multi-lectin affinity chromatography and digested with trypsin. Resulting glycopeptides were further enriched with HILIC SPE before LC-MS/MS analysis. *B*, bar plot showing the number of glycospecies identified from 30 brain samples. Technical triplicates of two normal brain samples are also included. *C*, overlap of glycospecies from normal, asymptomatic, and asymptomatic AD brains. Here, the glycospecies from all samples were pooled into the corresponding sample type. *D*, overlap of glycoproteins and glycopeptides identified from this work and those reported by Zhang *et al*. (20), which involved PNGase F–treated samples. AD, Alzheimer's disease; HILIC, hydrophilic interaction chromatography.

Total proteins from 30 human brain samples were extracted. A focused proteome database was generated (supplemental Table S5). The glycoproteins were enriched with multi-lectin affinity chromatography and subsequently digested with trypsin. The resulting intact glycopeptides were purified with HILIC SPE and analyzed with LC-MS/MS (Fig. 1*A*). A total of 303 glycoproteins were identified from 2035 unique glycopeptides, 580 N-linked glycosylation sites, and 124 glycans, making up a total of 1901 glycoforms (that is, if a single glycosylation site was identified with three different glycans, they are counted as three glycoforms) that were identified from all samples (Figs. 1*B*, S5, and supplemental Tables S6–S8). It should be noted that a single glycoform may arise from different-sized glycopeptides that share a single glycosylation site. Stringent criteria including manual validation were employed to filter the intact glycopeptide results for the final data set after Byonic analysis. Additionally, Neu5Gc-modified peptides were removed from the final dataset since previous reports showed that the monosaccharide is not present in human brains as well as the lack of enzyme to synthesize the monosaccharide (50, 51). Manual validation revealed that some of the annotated spectra with Neu5Gc-modified glycans contain diagnostic peaks for Neu5Ac at *m/z* 274.092 and *m/z* 292.103 but are not annotated as Neu5Ac (52). Additional searches with a glycan database without Neu5Gc showed that these spectra did not match with any glycopeptides from the database, which could be due to an unexpected modification on the glycopeptides.

The majority of the glycospecies, that is, glycoproteins, glycopeptides, glycosylation sites, and glycans, were identified commonly among the three brain types (Fig. 1*C*). Technical replicates were performed with two normal brain samples, that is, samples 170 and 363, denoted as replicates I, II, or III, where the brains were processed, enriched, and analyzed with MS separately. The overlap of glycoproteins, unique glycopeptides, glycosylation sites, glycans, and glycosylation sites with different glycans is acceptable among the three technical replicates (supplemental Fig. S6). There are also some runs where the coverage is lower than the others, including samples 363I, 223, 321, and 231. SDS-PAGE results showed that proteins were enriched from these samples (supplemental Fig. S7). We considered these as outliers and excluded them from some analysis when specified. On average, excluding low-coverage runs, we observed 138 glycoproteins, 644 unique glycopeptides, 242 unique N-linked glycosylation sites, 68 glycans, and 606 N-linked glycosylation sites with different glycans from each brain sample.

Compared to proteins annotated as being associated with AD on UniProt, cathepsin D, disintegrin and metalloproteinase domain-containing protein 10 (ADAM10), and sortilin-related receptor (SORL1) were determined to be glycosylated in our work. Tau and amyloid beta were not detected despite reports that showed that the proteins can be glycosylated, which could be due to their low abundance or the solubility of the plaques and tangles (17). Previously, Zhang *et al*. reported the de-N-glycosylated proteome of eight normal and eight age-matched AD brains (20). A comparison of our data with results obtained from that work showed a high overlap with the glycosylation sites and glycoproteins (gene name comparison) (Fig. 1*D*). It is expected that the coverage from this work would be lower due to the analysis at the intact glycopeptide level, which required a longer instrumentation time and more information to obtain the intact glycopeptide identification. Furthermore, the same glycopeptides may contain different glycans obtained from this work. Nevertheless, 183 glycopeptides and glycoproteins were specifically identified here, such as sushi domain-containing protein 2 (SUSD2), solute carrier family 12 member 6 (SLC12A6), and sodium channel subunit beta-3 (SCN3B).

**Fig. 2:**
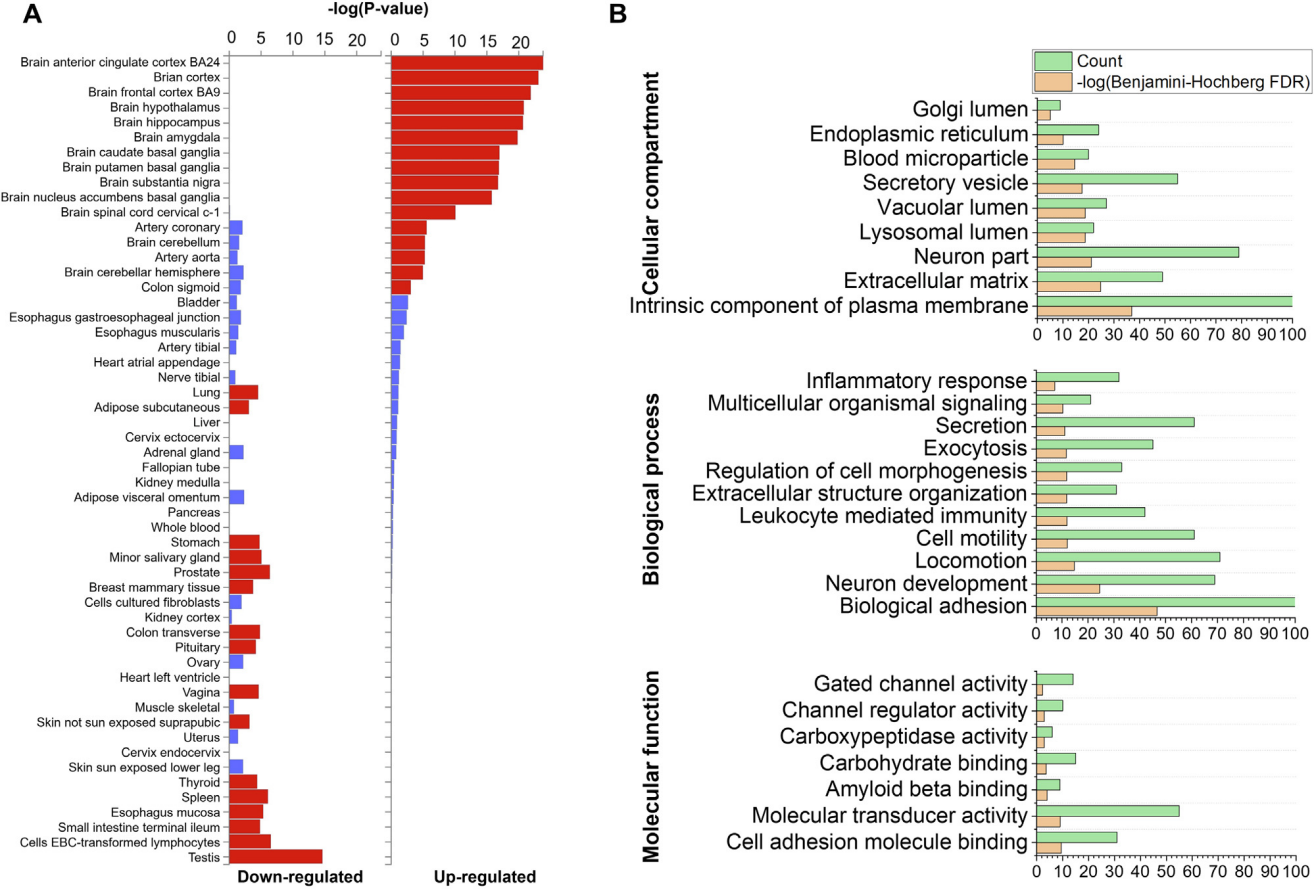
**Tissue expression and functional analysis.***A*, tissue expression of all glycoproteins identified based on FUMA GTEx V8 analysis against 54 tissues. *B*, Gene ontology clustering of identified glycoproteins based on cellular compartments (*top*), biological processes (*middle*), and molecular functions (*bottom*).

FUMA analysis for tissue expression revealed that the glycoproteins are enriched in different parts of the brain, including the cortex, hypothalamus, hippocampus, amygdala, basal ganglia, substantia nigra, spinal cord cervical, and cerebellum (Figs. 2*A* and S8) (31). As expected, proteins from other organs, such as the testis, spleen, and thyroid, were not enriched in our dataset. Gene ontology (GO) clustering based on cellular components showed that the identified glycoproteins are located on the plasma membrane, in the extracellular compartment or on neurons (supplemental Table S9). Both biological process– and molecular function–based GO clustering identified terms that correspond well with the functions of glycoproteins or proteins in neurons, such as biological adhesion and neuron development (Fig. 2*B*). Some glycoproteins were only identified in a specific sample type, that is, only in normal, asymptomatic AD, or symptomatic AD samples **(**supplemental Table S10). Clustering analysis revealed that these glycoproteins are enriched in the blood, such as complement factor H (H factor 1) or complement C1q subcomponent subunit A found in the normal brain samples (supplemental Fig. S9). Nevertheless, these were only found in four samples, indicating that they could result from contamination during sampling. The brains also contain vasculature, and thus some of these proteins may also be blood specific.

### Distribution of N-Glycosylation on Human Brain Glycoproteins

**Fig. 3:**
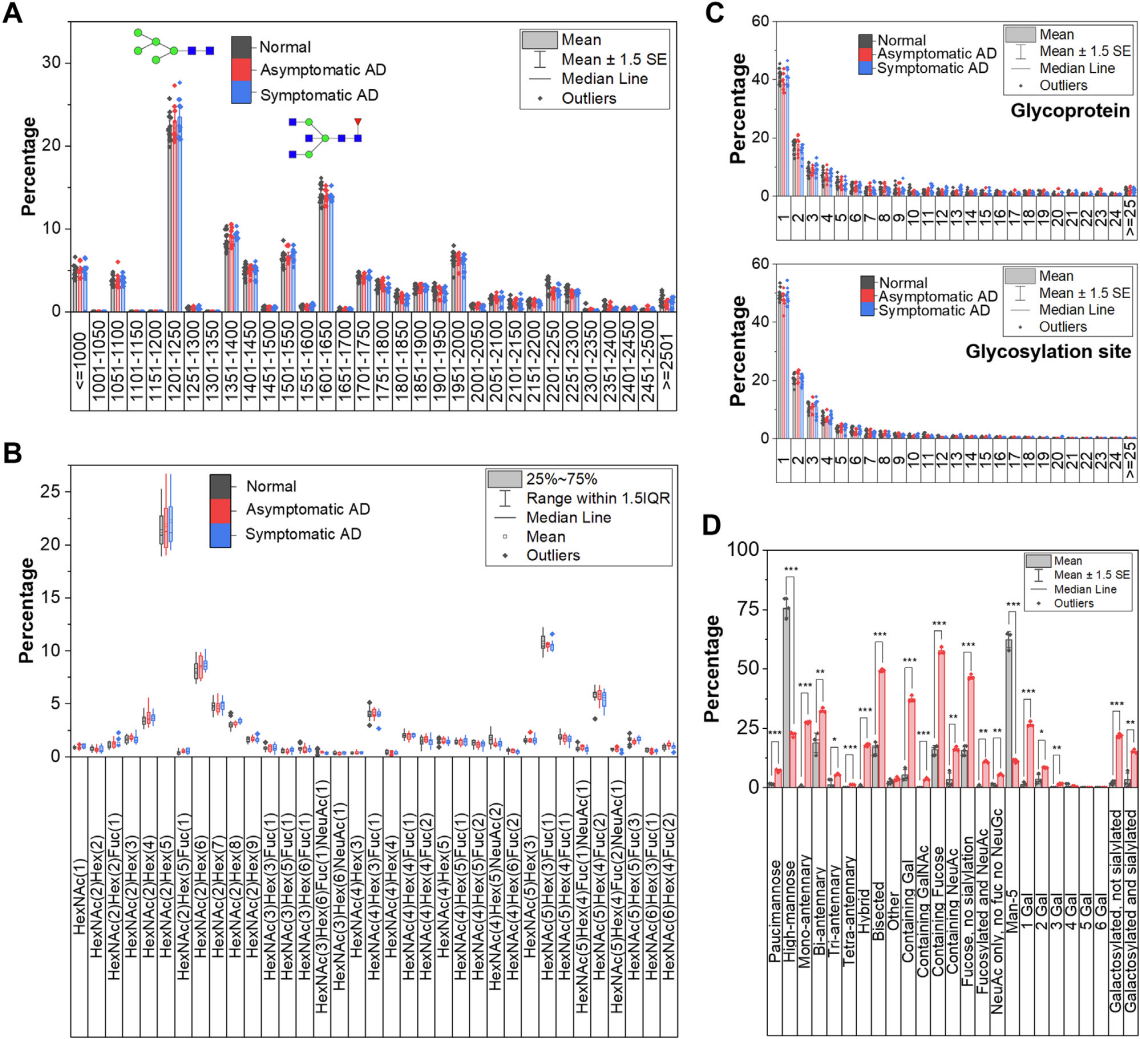
**Glycan distribution on identified glycoproteins.***A*, distribution of glycan molecular weight on unique glycopeptides from three sample types (*i.e.*, if the same glycan was identified on three different glycopeptides, the frequency is counted as three). The x-axis shows the molecular weight range in Da. Putative structures of the two most frequent glycans are shown. *B*, distribution of glycan occurrence frequency on unique glycopeptides from three sample types. Similar to the previous figure, if the same glycan was identified on three different glycopeptides, the frequency is counted as three. The x-axis shows glycan composition. Only glycoforms that were identified across all individual samples are shown (except for those with low coverage). *C*, number of different glycoforms on a protein or a glycosylation site. If the same glycan is found on three different glycosylation sites, it is counted as three. *D*, types of glycans present on glycoproteins with a single (*gray*) or multiple (*red*) glycoforms. *t* test was performed with Benjamini-Hochberg FDR correction. ∗, ∗∗, and ∗∗∗ show *p* < 0.05, *p* < 0.01, and *p* < 0.001, respectively. FDR, false discovery rate.

Byonic search resulted in >100 N-glycan compositions assigned to >500 glycosylation sites. We first examined the consensus motifs for N-linked glycosylation on the identified glycopeptides. As expected, both serine and threonine were enriched against the human proteome background at the third position of the sequence (supplemental Figs. S10 and S11). The majority of the glycopeptides contain the N-!P-T sequence with a higher proportion compared with the N-!P-S sequences, which has been reported previously (53). There is no statistical difference in the motif distribution among the three sample types. The glycans detected are considered as having a low molecular weight (MW < 2500 Da), in contrast to glycans found in other organs where the molecular weight is generally higher (Fig. 3*A*) (54). The most abundant glycans fall within the 1201 to 1250 MW range, which contribute to over 20% of the total glycoforms detected from the samples based on the glycan composition. Upon closer inspection, the majority of glycans in this MW range are from HexNAc(2)Hex(5), which is assigned as the Man_5_GlcNAc_2_ (Man5) structure (details for glycan type assignment are described in the next section) (Fig. 3*B*). A previous report employing MALDI-based glycomics also confirmed the high abundance of Man5 from both human and mouse brains compared with other glycans (32). The second most frequent glycan is HexNAc(5)Hex(3)Fuc(1), assigned as a biantennary glycan with a bisecting GlcNAc and core fucosylation. Other common glycans include several high-mannose, complex, and hybrid N-glycans with different degrees of fucosylation on glycopeptides were identified across the tissues (Fig. 3*B*). When considering the distribution based on the overall glycoforms, there is no statistical difference in the glycan molecular weight or glycan distribution among the different sample types.

Remarkably, the majority of the identified glycoproteins contained only one glycoform (Fig. 3*C*, top), that is, one glycan on a single glycosylation site within the protein. By contrast, a number of other glycoproteins have multiple glycans at each site. An example is neurofascin, which has 67 different glycoforms on 13 glycosylation sites (pooled from all samples). Most of the glycosylation sites also contained only one glycoform (Fig. 3*C*, bottom). The glycosylation site with the highest number of different glycans attached is N111 of CD47, which was identified with 36 different glycans. CD47 is a cell adhesion protein and a receptor for thrombospondin 1. The glycoprotein has six putative N-glycosylation sites, two of which were identified in this work including N111 and N73. In contrast to site N111, N73 was only identified with nine glycans while having similar solvent accessibility predicted by NetSurfP (relative surface accessibility of 0.396 for N73 and 0.341 for N111). The number of glycoforms in proteins/peptides does not appear to be statistically different among the three sample types.

Correlation between glycosylation site properties and the efficiency of protein glycosylation has been reported previously (55, 56). We further investigated the properties of these glycoproteins by separating them into proteins with only one glycoform and those with 10 or more glycoforms. First, we compared the number of N-!P-S/T sequences in glycoproteins with only one glycoform and those with 10 or more glycoforms. On average, glycoproteins identified with only one glycoform have seven N-!P-S/T sequences, while those with 10 or more glycoforms have, on average, 10 N-!P-S/T sequences with a statistical difference (supplemental Fig. S12*A*). NetSurfP 1.1 was then employed to determine the solvent accessibility of these asparagine residues with the N-!P-S/T sequence, which revealed that 16% of asparagine with the consensus sequence on proteins with one glycosylation site are buried, compared to 9% of those with 10 or more sites (supplemental Fig. S12*B*). Lastly, we looked into the glycans identified on these glycoproteins. The frequency of all glycopeptides with different glycoforms is significantly different for almost all glycan types except for those assigned as other, that is, the composition cannot be defined and those with three to six galactose residues (Fig. 3*D*). For example, for those with only one glycoform, high-mannose glycans were mainly present (75%), while for those with 10 or more glycoforms, only 22% of the total glycans are high-mannose.

### Alteration of Protein Glycosylation in Normal, Asymptomatic, and Symptomatic AD Brains

Once the glycosylation information had been collected, we investigated the different N-glycoproteome landscapes of these three sample groups - normal, asymptomatic, and symptomatic AD brains. Previously, multi-layered proteomics and glycoproteomics studies have shown that the protein and glycoprotein abundance of normal or disease tissues can be grouped into the corresponding phenotype based on their expression profiles (57, 58). With the qualitative data obtained, we first generated a heat map with unsupervised hierarchical clustering of the glycan composition on each glycosylation site of the proteins (supplemental Fig. S13). Apart from some glycosylation sites that contain glycans with the same composition across the different samples/sample types, we also observed different glycosylation extents at other glycosylation sites. As expected, we did not observe any clusters that separated the samples into normal, asymptomatic AD, or symptomatic AD types from our qualitative analysis. One clear distinction is the separation of the samples with low coverage from other runs at the four bottom rows. Additionally, we pooled the same information from all samples into the same sample type and performed the clustering (supplemental Fig. S14). We did not observe significant differences between the three sample groups.

**Figure 4:**
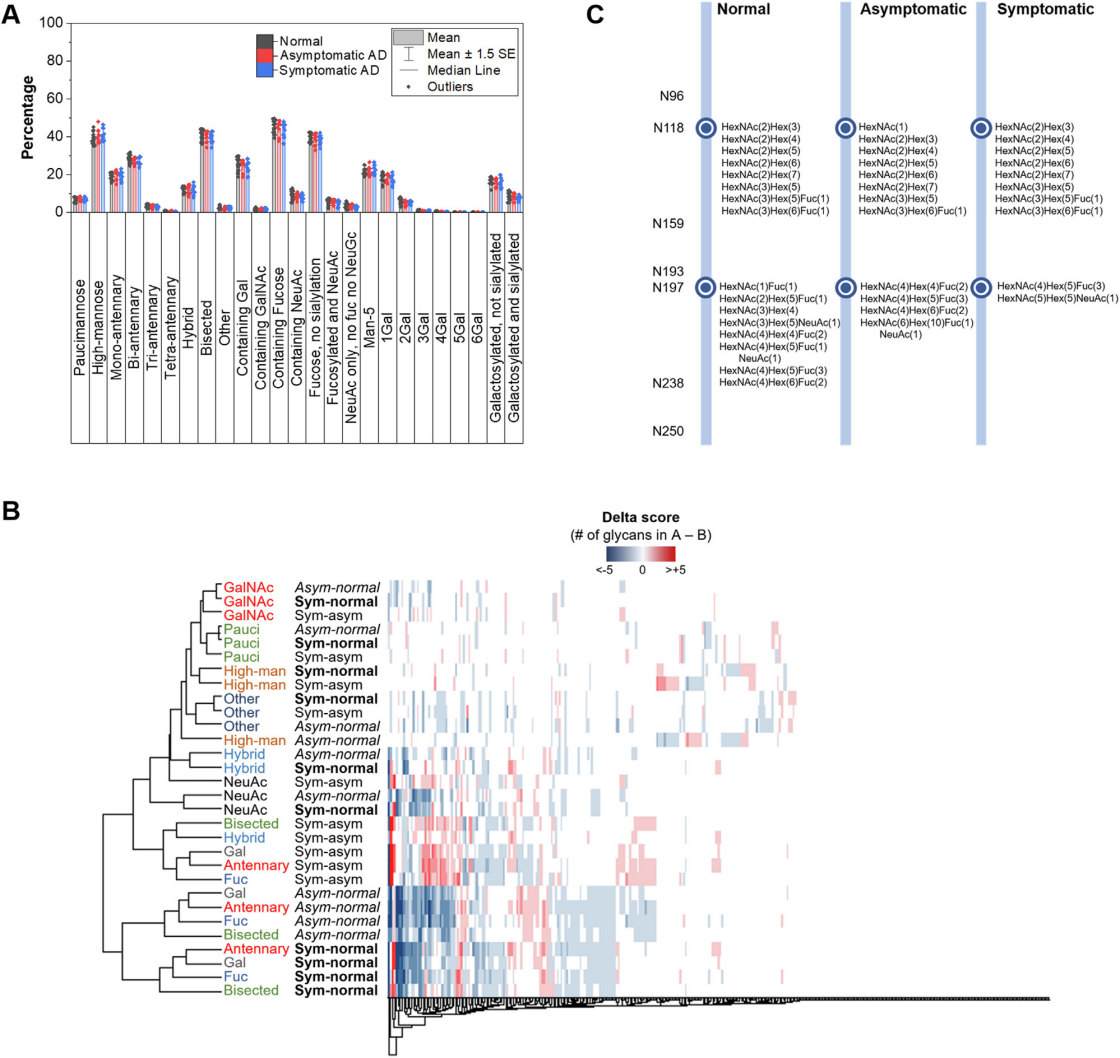
**Glycosylation alteration in normal, asymptomatic, and symptomatic AD brains.***A*, glycoform abundance in normal, asymptomatic, or symptomatic AD samples. Glycan type is determined based on the putative structure from the glycan composition determined by Byonic. *B*, heat map with hierarchical clustering of glycan types presented on glycosylation sites. Each column shows a specific glycosylation site, for example, O14594 + N122 and the difference in glycans for any feature pairs. The delta score refers to the number of glycans from the first sample type – the number of glycans in the second sample type. *C*, glycosylation of ATP1B2 in normal, asymptomatic, and symptomatic AD samples. Two of the seven identified glycosylation sites are shown. AD, Alzheimer's disease.

To gain more information about the glycosylation pattern in different sample groups, we reduced the glycosylation information to the glycosylation site level, which requires the analysis to encapsulate all glycan information at a specific glycosylation site before comparing the glycosylation among the samples. The glycans were first assigned into different glycan types with structural information according to a brain glycomics study by Williams *et al*. (32) Through an orthogonal method based on lectin blotting, the structures of these glycans were proposed in that work and can be used to explain the observations in ∼one-half of the glycans identified in this work. For the other ∼one-half of the composition without any structural information, we used GlyGen to predict and assign the structure to the one with the highest score. These putative glycan structures are described in supplemental Table S11. Similar to previous sections, if, for example, the same glycosylation site contains three different glycans, they are counted as having three glycoforms. The glycan types include paucimannose, high-mannose, mono-, bi-, tri-, and tetra-antennary, hybrid, bisected, galactosylated, GalNAcylated, fucosylated, and a combination of any monosaccharides (Fig. 4*A*). These analyses revealed that the majority of glycoforms are fucosylated, averaged at 44%, excluding low coverage runs. The majority of these are also fucosylated without any sialylation. The second most abundant glycosylation type is high-mannosidic (40%), which resulted from the Man5 glycan. Bisection is also a prominent feature of human brain complex-type N-glycans with an occurrence frequency of 40%. Galactosylation was also found in 25% of the glycoforms, with the majority containing only one galactose residue and a diminishing level up to as many as five residues. Sialylation, surprisingly, is present in a very low frequency (9%), unlike the glycome from other organs and tissues where the sialic acid is a major constituent in N-glycans, as we observed for N-glycans derived from human lungs, where ∼75% of the N-glycans carried at least one sialic acid (59). While the average glycan distribution from samples of a particular sample type showed an increase or decrease in glycosylation, such as the decrease of galactosylation in asymptomatic and symptomatic AD brains compared with normal brains, the difference is not statistically significant.

We performed a hierarchical clustering analysis of the glycosylation sites with the glycan type information for each site (supplemental Fig. S15). The same glycan types in normal, asymptomatic, and asymptomatic AD generally cluster together in the heat map. It also shows a preference for a specific glycan type on a particular glycosylation site, such as the presence of high-mannose glycans in all normal, asymptomatic, and symptomatic AD, without or with a minimal number of other types of glycosylation such as complex and hybrid glycans. Glycosylation is also more similar among the same glycan type in all normal, asymptomatic AD, and symptomatic AD and also observed in multiple glycan features, such as glycans with a bisecting GlcNAc, with galactose, with high-mannose, except for those with fucose and branched N-glycans (supplemental Fig. S15).

To further compare the difference in protein glycosylation among the sample types, we calculated a delta score, which shows the difference in the glycan types between any two different sample types (supplemental Table S12). Here, the glycans on each site from all brain tissues within the same sample type were pooled, the glycan types were assigned, and the difference was calculated (can also be considered as the difference in the number of glycan types for two sample types shown in the heat map of Fig. S15). The heat map showed that for each glycosylation site, there are changes in protein glycosylation types, either an increase or decrease in glycosylation at a specific site in ∼two-third of the glycosylation sites (Fig. 4*B*). Approximately, one-third of the glycosylation sites have the same glycosylation profile, which is similar to results from cerebrospinal fluids of AD patients (27). From the delta score, we determined the changes in protein glycosylation across the glycosylation sites for each glycan feature or at each glycosylation site for different sample types. When comparing different sample types (normal, asymptomatic AD, and symptomatic AD) for the collective changes in glycosylation across different glycosylation sites (that is, the total difference was added up), we observed changes in particular glycan features between any two sample types (supplemental Table S13). We observed a decrease in galactosylation, fucosylation, bisection, and the number of antennary glycans in asymptomatic and symptomatic AD samples, compared with normal brain samples. For asymptomatic compared to symptomatic AD samples, there are generally higher levels of galactosylation, fucosylation, bisection, and the number of antennary and hybrid glycans. In one example, sodium/potassium-transporting ATPase subunit beta-2 (ATP1B2) was found to be glycosylated in all AD sample types at seven glycosylation sites (Fig. 4*C*). Two of the seven sites are shown with different glycosylation changes across the sample types. For N118, the glycans are similar across the samples with the total site delta score of 0 (supplemental Table S12). The glycans are paucimannosidic, high-mannosidic, or hybrid with one antenna and core fucosylation. The HexNAc(3)Hex(6)Fuc(1) glycan that was identified in all sample types also contains a terminal galactose residue. All are not sialylated. On the contrary, N197 has a delta score of -43, showing changes in the glycans identified in the samples. HexNAc(4)Hex(5)Fuc(3), a complex glycan assigned with two antennae, core fucosylation, and two terminal galactose residues, was identified in all sample types. Another glycan identified in the symptomatic samples is HexNAc(5)Hex(5)NeuAc(1), which is a complex glycan with two antennae, a bisecting GlcNAc, and two terminal galactose residues. This stands in contrast to the normal samples, where several hybrid glycans were identified, such as HexNAc(3)Hex(5)NeuAc(1) or HexNAc(4)Hex(5)Fuc(1)NeuAc(1).

A similar comparison was also performed at each glycosylation site in order to determine the sites with the most changes in protein glycosylation. Here, the changes in glycosylation on the same site across different sample comparisons were calculated. The site with the highest overall increase in glycosylation is N234 of neurofascin (supplemental Table S14). Note that two, one, and seven glycan compositions were identified on this site, for the three sample types. The site with the highest decrease in glycosylation is at N330 of versican core protein (VCAN); a total of 23, 18, and 10 different glycans were identified on this site in normal, asymptomatic, and symptomatic AD samples, respectively (supplemental Table S14).

Nevertheless, ∼one-third of the glycosylation sites have a similar glycosylation profile (Fig. 4*B*). When comparing the parent glycoproteins with those with altered glycosylation profiles, the overlap is not high (supplemental Fig. S16). The 63 proteins with exclusively no changes in their glycosylation profile are involved in the regulation of sodium ion export across plasma membrane (*p* = 2.14E-4), heterotypic cell-cell adhesion (*p* = 1.47E-5), neuron projection regeneration (*p* = 2.46E-4), and positive regulation of phagocytosis (1.34E-4) based on GO biological processes. When examining the glycan types on these glycoproteins with no glycosylation alteration, the majority of glycans are high-mannosidic (51%) with some fucosylation (13%), bisection (11%), and antennary (14%), while for all samples, high-mannose is present at 40%, fucosylation at 44%, bisection at 40%, and antennary at 51% (supplemental Fig. S17). Note that the actual glycans that were compared might not have the same structure/composition but are of the same glycan type, for example, both Man5 and Man6 are considered as high-mannosidic glycans. While these have different compositions, they are considered as having the same glycan type. Thus, by this consideration, if they are present on the same glycosylation site in different sample types, the delta score will be zero.

Results were further combined with the glycosylation profile for each protein and compared the profiles between different sample types, giving the glycosylation difference at the protein level. Similar to results at the glycosylation site level, we still observed changes in glycosylation such as the decrease in fucosylation or galactosylation in symptomatic or asymptomatic AD samples compared with normal samples (supplemental Fig. S18).

### Glycosylation in Different Cellular Compartments

**Fig. 5:**
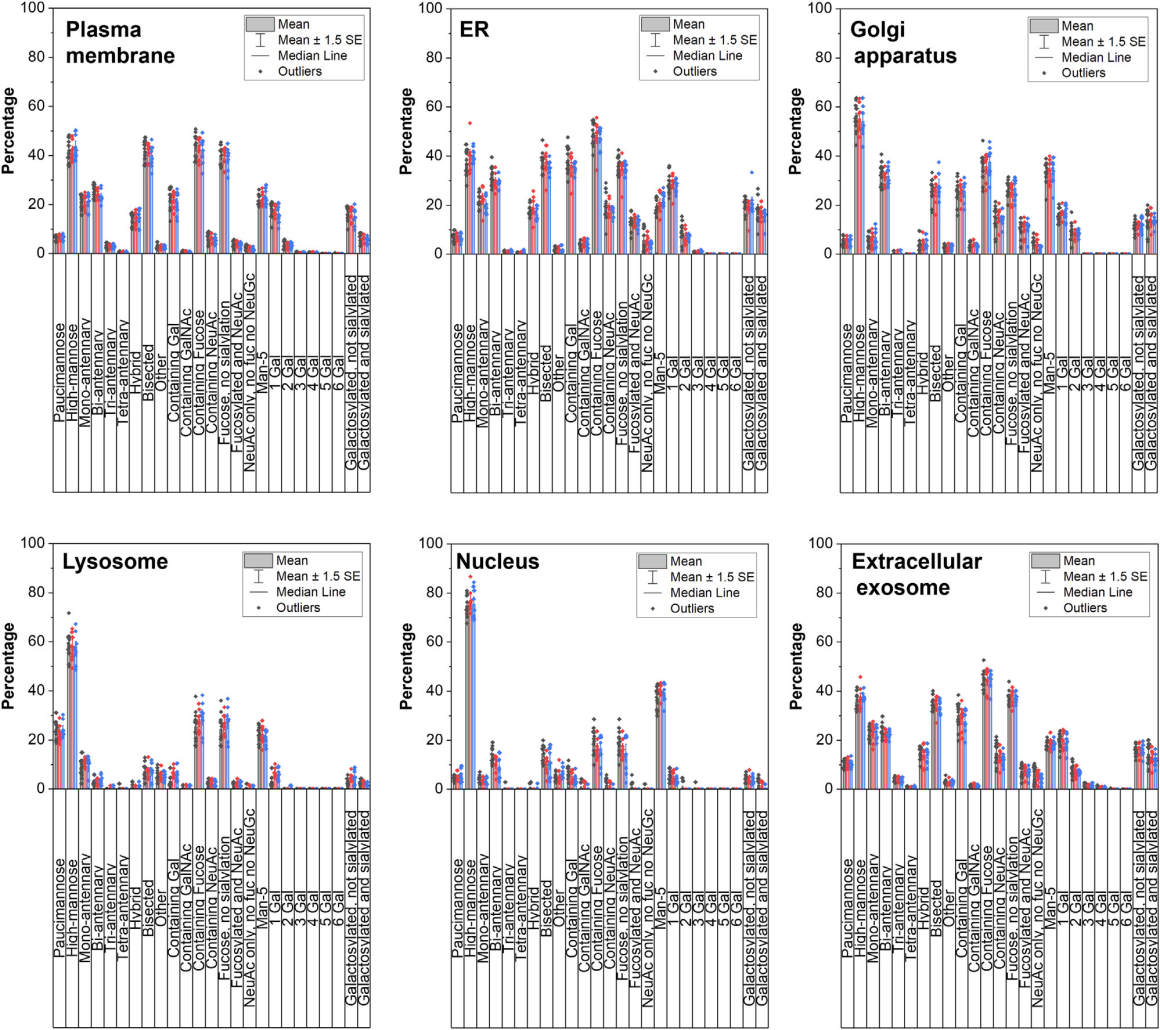
**Glycosylation in different cellular compartments.** Distribution of protein glycosylation and glycan types in six subcellular compartments. Subcellular location information was extracted from gene ontology at the protein level. One protein could be assigned to more than one cellular compartment.

We next investigated the glycosylation in different cellular compartments among the normal, asymptomatic, and symptomatic AD brains. Subcellular localizations that are predicted, including the plasma membrane, ER, Golgi apparatus, lysosome, nucleus, and extracellular exosome, of the identified glycoproteins were extracted from their cellular compartment GO. Hence, one glycoprotein could be present and analyzed in several compartments. Note that the localization information is extracted from GO data, and thus these glycoproteins may not be truly localized in the nucleus. In all compartments, the majority of the glycoproteins identified are modified with high-mannosidic glycans, except for the ER where the majority are fucosylated glycans (Figs. 5 and S19). In the Golgi apparatus, the percentage of high-mannosidic glycans is higher than the plasma membrane and the ER (56% vs 40%) but is still lower than the nucleus (76%). Glycoforms found in the lysosome and nucleus are more similar than the other four compartments, that is, low levels of hybrid, bisected, antennary structures. The lysosome also has the highest percentage of paucimannosidic glycans (24%) than other compartments (<10%). The distribution of glycoforms in the lysosome and the nucleus glycoforms are also more similar than other compartments, that is, low levels of hybrid, bisected, or antennary structures. The nucleus also has the highest percentage of high-mannosidic glycans (76% compared with <50% in other compartments). The distribution profiles in the plasma membrane, ER, Golgi apparatus, and extracellular exosome are similar, which could be due to all being in the canonical signaling pathway. ANOVA showed that there is no statistical difference for each glycan type in each compartment between normal, asymptomatic, or symptomatic AD samples. However, the test showed that there is a statistical difference for different glycan abundance for all six subcellular compartments investigated.

The brain samples analyzed were also selected from both male and female patients. Reports have shown some correlation between sexes and the occurrence of AD, which could be due to the contribution from several factors such as longevity, hormones, inflammation and metabolism, genetics and epigenetics, and cognition (60). We performed similar analyses as those in previous sections, such as the distribution of glycans and glycoforms and glycan molecular weight (supplemental Figs. S20 and S21), except that the samples were also separated into male and female samples (total of six sample types: male or female of normal, asymptomatic, and symptomatic AD). While results are not statistically significant, we observed, for example, a higher percentage of high-mannosidic glycoforms in female asymptomatic AD samples or decreased in fucosylation in female asymptomatic AD samples compared with normal male samples (supplemental Fig. S20). Similar to Figure 4*B*, we also calculated the delta score and performed hierarchical clustering analysis of the glycan type distribution. While the difference among the samples does not reach statistical significance, we observed some changes such as the increase in bisection in symptomatic male samples compared with asymptomatic male and female and normal female samples (supplemental Fig. S22). Thus, the results indicate that future studies should incorporate many more male and female samples to address these potential differences.

## Discussion

Our results provide new information about the glycoproteome landscape of normal human brains and those from asymptomatic and symptomatic AD brains. Previous studies have focused on the analysis of a small number of glycoproteins that could be involved in AD or the analysis of released N-glycans from AD brains. Here, we present a large-scale, intact glycopeptide analysis and the investigation of brain N-glycosylation changes across the AD clinical spectrum. As glycoproteins are generally present in a low abundance in human cells, we employed multi-lectin chromatography to enrich glycoproteins from the brain lysates, followed by HILIC SPE for subsequent glycopeptide enrichment. The approach was optimized so that N-linked glycopeptides with multiple glycan types were effectively captured. The enriched glycopeptides were subsequently analyzed with LC-MS/MS. This approach resulted in the identification of 580 N-linked glycosylation sites and 124 glycan compositions on 2035 unique glycopeptides from 303 glycoproteins across the brain samples, making this the first global view of glycoproteins with intact glycans in human brains that consists of 1901 glycoforms. Most glycoproteins and glycopeptides were identified in all sample types. Functional analysis of identified glycoproteins showed that the glycoproteins are located on the plasma membrane, in the extracellular matrix, or the neuronal part. Only a minor contaminating portion of glycoproteins detected originated from the blood. The analysis also showed that brain glycoproteins function, as would be expected, in multiple biological pathways, including cellular adhesion, neuron development, molecular signal transduction, or carbohydrate binding. It should also be noted that we did not control for the influence of pre-analytical factors, such as postmortem intervals between death and sample collection or consider the use of RNA integrity number. However, if variations from pre-analytical factors did occur by chance, we would not have observed some statistical significance results such as the prominent presence of Man5 glycans in the brain. As we accumulated the data, we also observed this significance, which led us to employ this number of samples in our analysis. We also intended to profile the overall glycosylation profile of human brains in stages of AD and did not separate between white and gray matters. In future studies, this might shed additional light on differences in protein N-glycosylation AD brains as we investigate the glycosylation profiles in these brain layers or cells types.

Byonic search resulted in glycan compositions on the glycopeptides. We assigned a structure to these compositions based on a previous glycomics study of human brains (32, 61). The majority of the glycopeptides contain HexNAc(2)Hex(5), which is assigned as Man5 or are fucosylated. Many glycoproteins also contain a bisecting GlcNAc residue. Sialylation, however, only occurred in a small fraction of the N-glycans in glycopeptides/glycoproteins. The results are similar to the glycoproteome of brain samples from WT and AD mouse models (62).

Overall, our results agree with previous studies that investigated the human brain glycoproteome outside of the AD context. For example, the studies of Brown *et al*. identified high-mannosidic glycans as the largest glycan family in the brain (∼35%), while it is only present at ∼15% in the serum. HexNAc(2)Hex(5) and HexNAc(2)Hex(6) were found at 20% of the identification in the brain, and only a small portion of the glycopeptides identified are sialylated (<27%) compared with >57% in the serum (63). In studies by Gaunitz *et al*. who performed quantitative N-glycan profiling in the cortex and hippocampus of normal and AD brains, they observed that the majority are complex-type glycans (70%) that are decorated with fucose and high-mannosidic (20%), with a few glycans that contain sialic acid or fucose that differed between control and AD brains (64). In our results, HexNAc(2)Hex(5) and HexNAc(2)Hex(6) were found at 30%, sialylation only occurred in 9%, and 40% are high-mannosidic. Qualitatively, our results are in accordance with those prior studies, and any differences in the relative amounts of materials could be due to our specific enrichment strategy, as well as potential differences in the downstream analytical approaches.

In our studies on the transcriptome and N-glycans in the murine brain and human brain (32, 47, 65), the expression profiles of enzymes are consistent with the structures of the proposed glycans and their relative abundance. In terms of such predicted structures, based on the murine brain and human transcriptomic analyses, we can predict that in the human brain, low sialylation is partly due to low levels of expression of sialyltransferases that modify N-glycans in the frontal and cortical lobes of AD brains, as also observed previously (66). The presence of fucosylated and bisected N-glycans of abundance is consistent with high expression of specific fucosyltransferases and the bisecting N-acetylglucosaminyltransferase GnT-III, which has been noted previously in murine and human brains (32, 67, 68, 69, 70). In our analyses, we should note that our putative structural assignments are based on monosaccharide compositional analyses, and a single glycan composition may represent multiple glycan structures and linkages (71, 72).

When comparing the occurrence of individual N-linked glycosylation events, that is, comparing specific glycan composition on a particular site of proteins between samples, in all samples from the three sample types, there is no statistical difference among the samples. Such modifications also did not cluster into separate sample types. Taking advantage of the glycan type assignment, we compared the glycan types on glycoforms of glycopeptides from different samples. For example, instead of comparing whether HexNAc(2)Hex(5) is present on N223 of neuronal cell adhesion molecule, we compared the number of glycan types, such as paucimannosidic or high-mannosidic at a particular site between different sample types. When comparing the total glycoforms for each glycan type in the three sample types, there is no statistical difference between the samples. In one previous related study, Gizaw et al. investigated the glycomics profile of 12 AD and normal brain samples, including both male and female, and reported that the expression level of glycans in AD and normal brains are mostly similar except for the decrease in N-glycans in the frontal cortex of AD patients. There is no significant difference in most N-glycans in other cerebral cortices even though the abundances of high-mannose, core fucose, and bisecting GlcNAc appear to be somewhat lower in AD patients. This might arise from the downregulation of GnT-III and FUT8 in the brains but that remains to be studied. Nevertheless, the abundance of some glycans, for example, branched and bisected glycans, in serum and CSF increased significantly in AD patients (73). GnT-III expression is increased in AD brains, as well as glycans with bisecting GlcNAc. The upregulation of GnT-III due to Aβ treatment and decrease in Aβ in GnT-III–transfected cells showed that this could be the adaptive response from additional Aβ production (74).

Our studies revealed alterations between the three sample groups in protein glycosylation at the glycosylation site level, especially the changes in frequency of bisection, fucosylation, galactosylation, and the number of antennae, which differed by clinical stage. The biggest alteration is the increase in the number of antennae from asymptomatic to symptomatic AD samples. Note that a potential drawback of this approach is that the glycosylation information was pooled from all samples for a particular sample type, and thus not every sample may contain that glycoform. In a study by Fang *et al*., glycosylation changes in WT and AD mouse brains were quantified by glycoproteomics and lectin microarray and revealed the decreased binding of lectins to several glycan types, notably fucosylated and high-mannosidic glycans, in AD mice (62). These differences in glycosylation site utilization and structure could profoundly affect protein functions, but such specific relationships and consequences of these changes will be explored in the future.

It should be noted that we did not use any tissue quality metric and pre-analytical variation could exist. This could result in glycoprotein degradation or changes in protein abundance in the brain. Nevertheless, since the samples were obtained postmortem, it would be a parameter that is not possible to control, as well as other parameters such as comorbidity or medications. Postmortem interval has already been shown to minimally contribute to the first four principal components in protein abundance (75). RNA integrity number was not used as it is a measure of RNA integrity, not the protein abundance, and should not be correlated with glycoprotein abundance. Furthermore, it is not a predictor of what will pass the quality control. We believe that our sample size is large enough and should minimize the effect from postmortem interval. We did not select for gray or white matter in our study, as we aimed to perform a general analysis of the frontal cortex as a whole.

In a separate analysis, we investigated the O-glycoproteome of the brain samples. However, because the lectin enrichment strategies were designed and optimized primarily for N-glycans, we could not obtain sufficient information at this stage about the O-glycoproteome. Released O-glycans have been analyzed in Parkinson’s disease, but an additional enzyme such as StcE or OpeRATOR, as well as an additional peptide enrichment method specific for O-glycopeptides, will aid future analyses of O-glycopeptides in neurodegenerative processes (76, 77, 78). O-GlcNAcylated proteins were also specifically enriched by WGA, and while silver-stained SDS-PAGE showed the presence of WGA-binding proteins (supplemental Fig. S23), we observed few O-GlcNAcylated peptides by MS after PNGase F treatment. Analysis of O-glycoproteins, using alternative enrichment strategies, will be the focus of future work.

In summary, we investigated the N-glycoproteomic landscape of normal, asymptomatic, and symptomatic AD brains using lectin enrichment, HILIC, and LC-MS/MS. While the current work showed qualitative analysis, future work will include quantitative analyses on abundance of glycoforms among a larger number of samples. These results will contribute to our knowledge of protein glycosylation in the brain and reveal the role(s) of N-glycoproteomic changes in AD. Of particular interest will be glycosylation changes that arise in asymptomatic AD samples to guide future research on biomarker and therapeutic development.

## Data Availability

The mass spectrometry proteomics data have been deposited to the ProteomeXchange Consortium *via* the PRIDE (79) partner repository with the dataset identifier PXD032219. ROSMAP resources can be requested at https://www.radc.rush.edu/. All other data are presented in the manuscript with no restrictions.

## Supplemental data

This article contains supplemental data.

## Conflict of interest

The authors declare that they have no conflicts of interest with the contents of this article.
