# Supplementary material for: Glycoproteomics Landscape of Asymptomatic and Symptomatic Human Alzheimer’s Disease Brain
Source: Mol Cell Proteomics. 2022 Oct 27;21(12):100433. doi: 10.1016/j.mcpro.2022.100433 (PMC9706167; doi:10.1016/j.mcpro.2022.100433)
Supplement: Supplemental Information [file mmc1.docx]

**Supplemental Information**

**Glycoproteomics Landscape of Asymptomatic and Symptomatic Human Alzheimer’s Disease Brain**

Suttipong Suttapitugsakul^1,2^, Kathrin Stavenhagen^1,2^, Sofia Donskaya^1^, David A. Bennett^3^,

Robert G. Mealer^4^, Nicholas T. Seyfried^5^, and Richard D. Cummings^1,6^

^1^Department of Surgery, Beth Israel Deaconess Medical Center, Harvard Medical School, Boston, MA, USA

^2^These individuals should be considered co-first authors

^3^Rush Alzheimer's Disease Center, Rush University Medical Center, Chicago, Illinois, USA

^4^Psychiatric and Neurodevelopmental Genetics Unit, Department of Psychiatry, Massachusetts General Hospital, Harvard Medical School, Boston, MA, USA

^5^Department of Biochemistry, Emory University School of Medicine, Atlanta, Georgia, USA

^6^**Corresponding author:** Richard D. Cummings, Ph.D., National Center for Functional Glycomics, Department of Surgery, Beth Israel Deaconess Medical Center, Harvard Medical School, CLS 11087, 3 Blackfan Circle, Boston, MA 02215, Tel: 1-617-735-4643, [rcummin1@bidmc.harvard.edu](mailto:rcummin1@bidmc.harvard.edu)

**Short title:** Glycoproteomics landscape of Alzheimer’s brain

**Keywords:** Glycoproteomics, Alzheimer’s disease, symptomatic, asymptomatic, lectin enrichment, N-linked glycosylation

**Supplemental Table Legends**

(Excel) **Table S1:** **Sample information.** Information on the 30 brain samples, including the sample number, age at death, APOE gene, Braak and CERAD scores, and AD diagnosis.

(Excel) **Table S2: Lectin and HILIC optimization**. Glycoproteomics results for the selection of lectins and the enrichment approach with and without HILIC.

**Table S3. Optimization of the enrichment of glycoproteins.** Glycoproteomics results from flow-through or eluates after lectin or HILIC enrichment.

(Excel) **Table S4: Protein amount optimization**. Glycoproteomics results for the selection of lectins and the enrichment approach with and without HILIC. Summarized results are in **Table S3**.

(Excel) **Table S5: Proteins identified for the focused proteome databased.** Proteins identified from the cohort samples. These proteins were combined and used as a focused proteome database for glycoproteomics searches.

(Excel) **Table S6: Glycoproteins identified from normal brains.** The filtered glycoproteomics results obtained from Byonic of the 10 normal brain samples. Results are separated into different sheets by sample ID (see **Table S1**). Technical replicate runs are labeled with I, II, and III.

(Excel) **Table S7: Glycoproteins identified from asymptomatic AD brains.** The filtered glycoproteomics results obtained from Byonic of the 10 asymptomatic AD brain samples. Results are separated into different sheets by sample ID (see **Table S1**).

(Excel) **Table S8: Glycoproteins identified from symptomatic AD brains.** The filtered glycoproteomics results obtained from Byonic of the 10 symptomatic AD brain samples. Results are separated into different sheets by sample ID (see **Table S1**).

(Excel) **Table S9: Gene ontology analysis of identified glycoproteins.** Gene ontology results from FUMA analysis of all identified glycoproteins from all samples. Results are separated into GOs enriched from cellular compartments, biological processes, and molecular functions.

(Excel) **Table S10: Glycoproteins identified in a particular sample type.** Glycoproteins identified specifically in normal, asymptomatic, or symptomatic AD brains.

(Excel) **Table S11: Putative glycan structures.** Glycan compositions obtained from Byonic were assigned a structure according to a study by Williams et al., and the structural information, such as glycan type (paucimannose, high-mannose, etc.) or features (bisected, antenna, etc.) are described. For about half of the glycans without any glycan information, GlyGen was used to assign the structures.

(Excel) **Table S12.** **Difference in glycan types between different sample types across the glycosylation sites.** The sum difference is calculated from the total difference in glycan types among the sample pairs.

**Table S13. Delta score.** Difference in number of glycan features for the three sample types. Data shown here are also plotted in **Figure 4B**.

**Table S14.** **Collective changes in glycosylation at the site level.** The sum delta scores of the glycosylation sites with the highest increase in glycan features (N234 of NFASC) and the highest decrease (N330 of VCAN).

**Supplemental Tables**

**Table S3.** **Optimization of the glycoprotein enrichment.** Glycoproteins were enriched using multi-lectin affinity chromatography. The flow through was subjected to a second round of enrichment to evaluate lectin capacity. Lectin enrichment fractions were further enriched for glycopeptides by HILIC SPE prior to LC-MS analysis. HILIC flow through and wash solutions were also analyzed to evaluate HILIC capacity. Number of all identified glycopeptides was used for comparison.

| HILIC fraction | Lectin enrichment fraction | Lysate input (micrograms) | Unique glycopeptides | Total glycopeptides |
| --- | --- | --- | --- | --- |
| HILIC elution | 1st elution | 660 | 548 | 1198 |
|  | 1st elution | 830 | 930 | 1964 |
|  | 1st elution | 1000 | 882 | 1745 |
|  | 1st elution | 1250 | 853 | 1698 |
| HILIC elution | 2nd elution | 660 | 55 | 156 |
|  | 2nd elution | 830 | 93 | 274 |
|  | 2nd elution | 1000 | 123 | 341 |
|  | 2nd elution | 1250 | 141 | 374 |
| HILIC elution | 1st wash | 660 | 5 | 10 |
|  | 1st wash | 830 | 5 | 12 |
|  | 1st wash | 1000 | 7 | 15 |
|  | 1st wash | 1250 | 4 | 8 |
| HILIC wash and flow through | 1st elution | 660 | 15 | 40 |
|  | 1st elution | 830 | 20 | 43 |
|  | 1st elution | 1000 | 27 | 71 |
|  | 1st elution | 1250 | 16 | 35 |
| HILIC elution | 1st elution | 250 | 239 | 630 |
|  | 1st elution | 500 | 497 | 1145 |
|  | 1st elution | 660 | 361 | 865 |
|  | 1st elution | 830 | 403 | 1025 |
| HILIC elution | 2nd elution | 250 | 20 | 44 |
|  | 2nd elution | 500 | 42 | 119 |
|  | 2nd elution | 660 | 32 | 78 |
|  | 2nd elution | 830 | 45 | 128 |

**Table S13.** **Difference in glycan types between different sample types across the glycosylation sites.** The sum difference is calculated from the total difference in glycan types among the sample pairs.

| **Glycan feature** | **Comparison** | **Sum difference** | **Standard deviation** |
| --- | --- | --- | --- |
| Antenna | Sym vs asym | 29 | 1.01 |
| Fucose | Sym vs asym | 22 | 0.92 |
| Bisected | Sym vs asym | 22 | 0.72 |
| Galactose | Sym vs asym | 21 | 0.78 |
| Hybrid | Sym vs asym | 20 | 0.48 |
| High-Man | Sym vs asym | 14 | 0.42 |
| NeuAc | Sym vs asym | 8 | 0.52 |
| Pauci | Sym vs asym | 4 | 0.26 |
| GalNAc | Sym vs asym | 1 | 0.29 |
| Pauci | Sym vs normal | -2 | 0.28 |
| Pauci | Asym vs normal | -6 | 0.26 |
| High-Man | Sym vs normal | -7 | 0.33 |
| Hybrid | Sym vs normal | -13 | 0.59 |
| GalNAc | Sym vs normal | -18 | 0.32 |
| Other | Sym vs normal | -18 | 0.34 |
| GalNAc | Asym vs normal | -19 | 0.28 |
| High-Man | Asym vs normal | -21 | 0.41 |
| Other | Sym vs asym | -29 | 0.40 |
| Hybrid | Asym vs normal | -33 | 0.47 |
| Other | Asym vs normal | -50 | 0.46 |
| NeuAc | Sym vs normal | -52 | 0.73 |
| NeuAc | Asym vs normal | -60 | 0.67 |
| Bisected | Sym vs normal | -64 | 0.88 |
| Galactose | Sym vs normal | -84 | 1.01 |
| Bisected | Asym vs normal | -86 | 0.76 |
| Fucose | Sym vs normal | -104 | 1.16 |
| Galactose | Asym vs normal | -105 | 0.95 |
| Antenna | Sym vs normal | -107 | 1.24 |
| Fucose | Asym vs normal | -126 | 1.02 |
| Antenna | Asym vs normal | -136 | 1.15 |

**Table S14.** **Collective changes in glycosylation at the site level.** The table shows the sum delta scores of the glycosylation sites with the highest increase in glycan features (N234 of NFASC) and the highest decrease (N330 of VCAN).

|  | **Glycan** | **Sample type** | **NFASC (O94856-3)** | **VCAN (P13611-3)** |
| --- | --- | --- | --- | --- |
|  |  |  | **N234** | **N330** |
| **Delta score** | GalNAc | Normal | 0 | 0 |
|  |  | Asymptomatic AD | 0 | -2 |
|  |  | Symptomatic AD | 0 | -2 |
|  | Galactose | Normal | 0 | -3 |
|  |  | Asymptomatic AD | 5 | -9 |
|  |  | Symptomatic AD | 5 | -6 |
|  | Fucose | Normal | -1 | -5 |
|  |  | Asymptomatic AD | 5 | -13 |
|  |  | Symptomatic AD | 6 | -8 |
|  | NeuAc | Normal | 0 | -2 |
|  |  | Asymptomatic AD | 2 | -6 |
|  |  | Symptomatic AD | 2 | -4 |
|  | Pauci | Normal | 0 | -1 |
|  |  | Asymptomatic AD | 0 | -1 |
|  |  | Symptomatic AD | 0 | 0 |
|  | High-Man | Normal | 0 | 0 |
|  |  | Asymptomatic AD | 0 | 0 |
|  |  | Symptomatic AD | 0 | 0 |
|  | Hybrid | Normal | 0 | -3 |
|  |  | Asymptomatic AD | 1 | -5 |
|  |  | Symptomatic AD | 1 | -2 |
|  | Bisected | Normal | -1 | -3 |
|  |  | Asymptomatic AD | 4 | -7 |
|  |  | Symptomatic AD | 5 | -4 |
|  | Antenna | Normal | -1 | -4 |
|  |  | Asymptomatic AD | 5 | -12 |
|  |  | Symptomatic AD | 6 | -8 |
|  | Other | Normal | 0 | 0 |
|  |  | Asymptomatic AD | 0 | 0 |
|  |  | Symptomatic AD | 0 | 0 |

**Supplemental Figures**

**
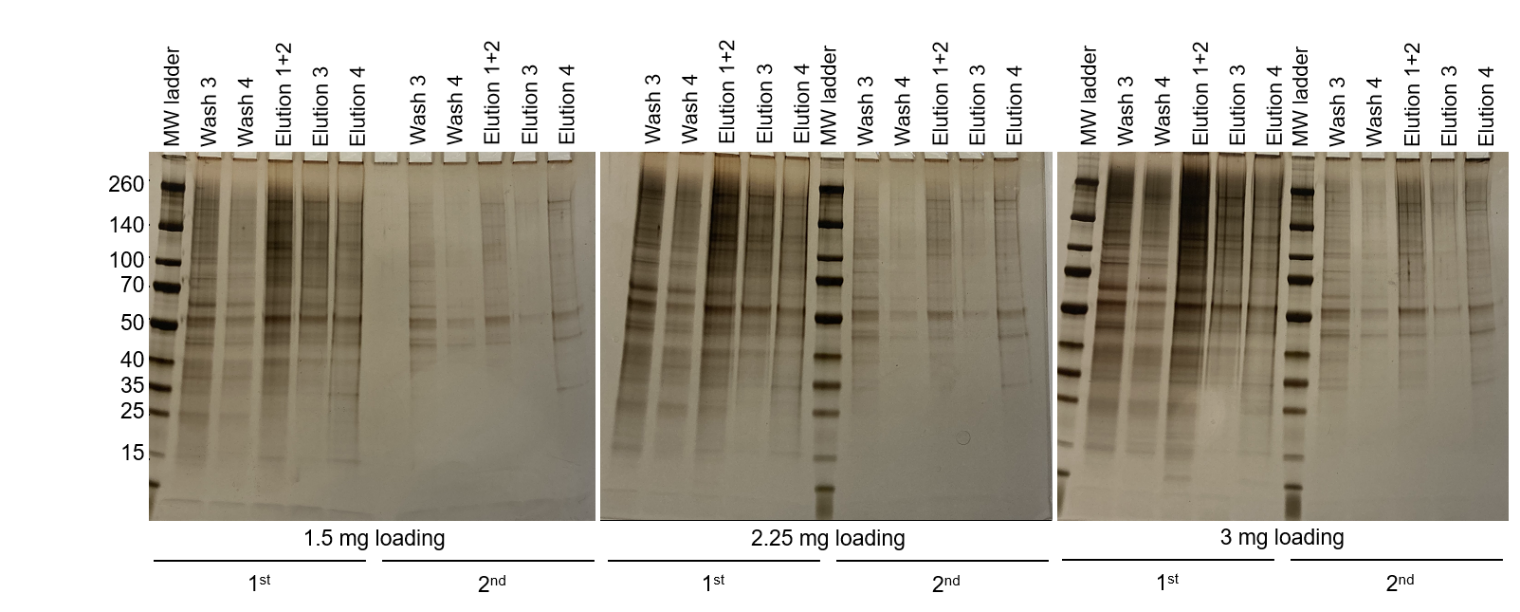
**

**Figure S1. Optimization of the enrichment of glycoproteins from mouse brains with Con A, RCA, and SNA.** After multi-lectin enrichment, proteins from the wash and elution solutions were separated by SDS-PAGE. Glycoproteins in the flow through were further re-enriched and separated (2^nd^). The silver-stained gels showed that most glycoproteins were effectively captured after the first round of enrichment.


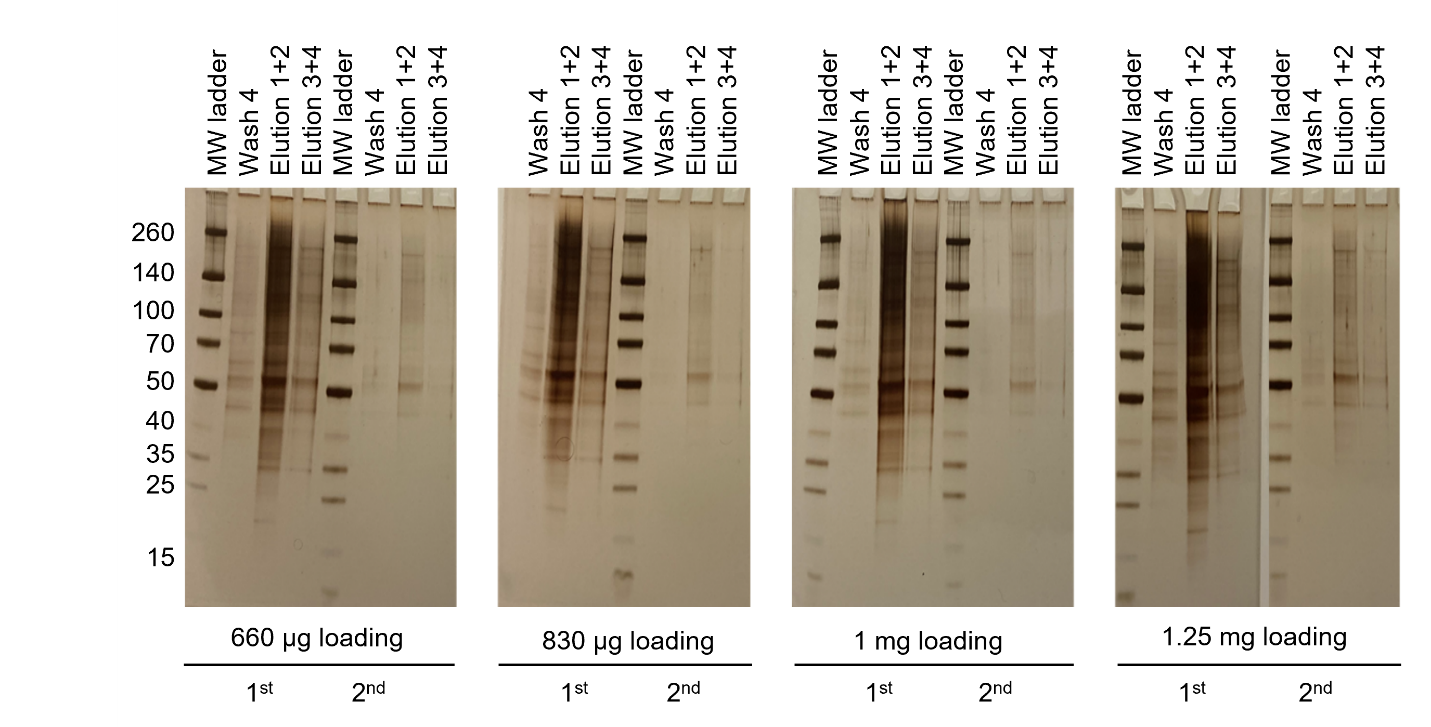


**Figure S2.** **Optimization of the enrichment of glycoproteins from mouse brains with ConA, RCA, SNA, AAL, and WGA.** Similar to Figure S1, after multi-lectin enrichment, proteins from the wash and elution solutions were separated by SDS-PAGE. Glycoproteins in the flow through were further re-enriched and separated (2^nd^). The silver-stained gels showed that most glycoproteins were effectively captured after the first round of enrichment.


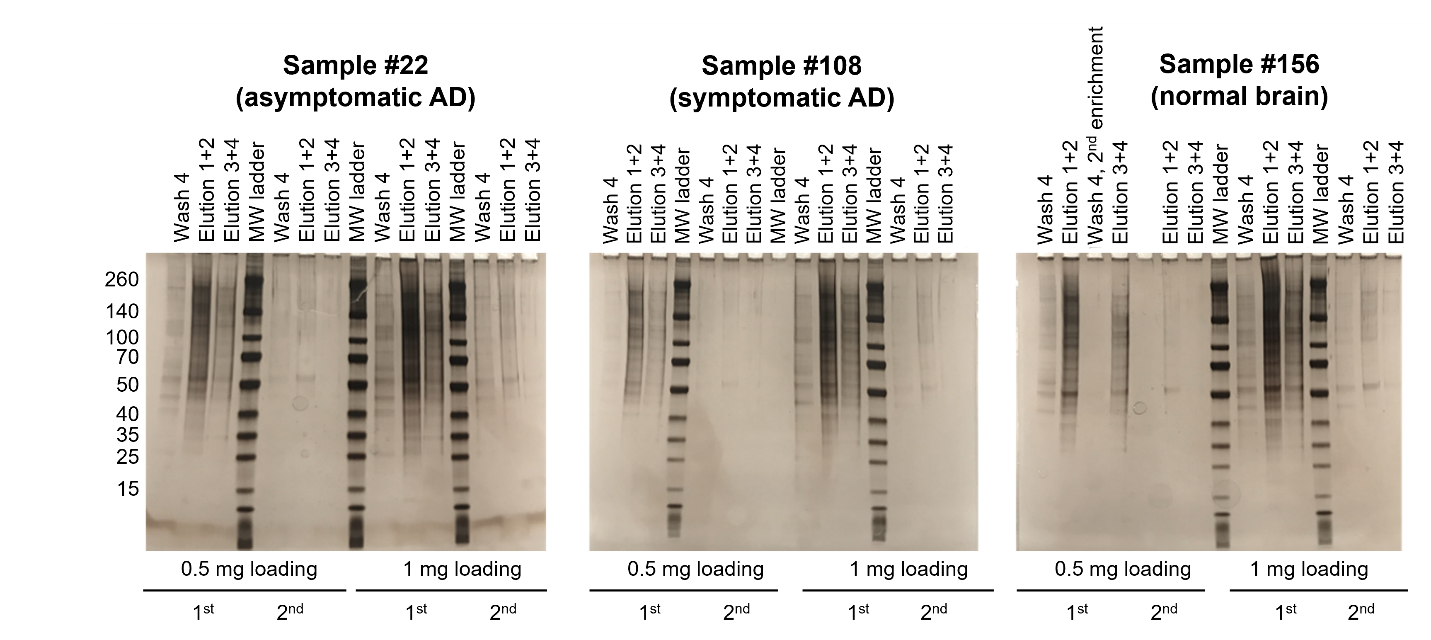


**Figure S3. Enrichment of glycoproteins from human brain samples.** Glycoproteins from normal, asymptomatic AD, and symptomatic AD human brain samples were enriched with multi-lectin chromatography and separated on SDS-PAGE. The gels were silver-stained for imaging.


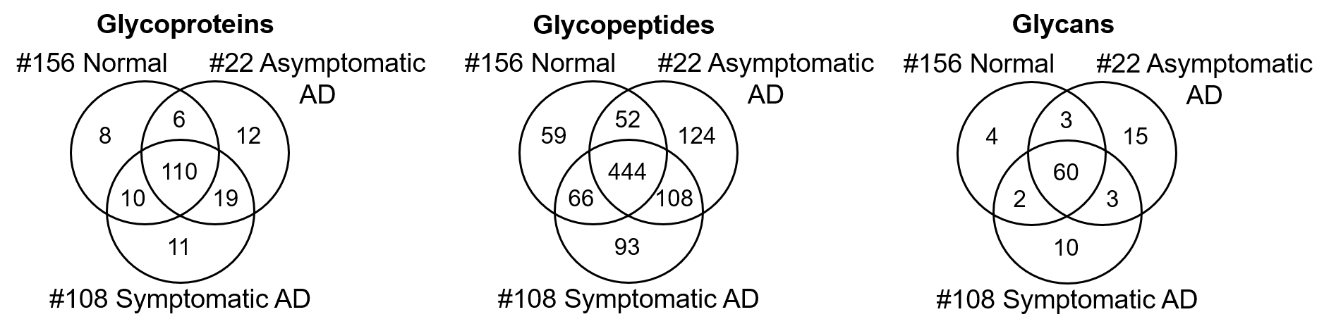


**Figure S4. Overlap of glycospecies from the trial experiment with three human brain samples.** The method combining multi-lectin enrichment and HILIC SPE was applied to three brain samples. Results showed that our method is effective for intact glycoproteomics analysis.

**
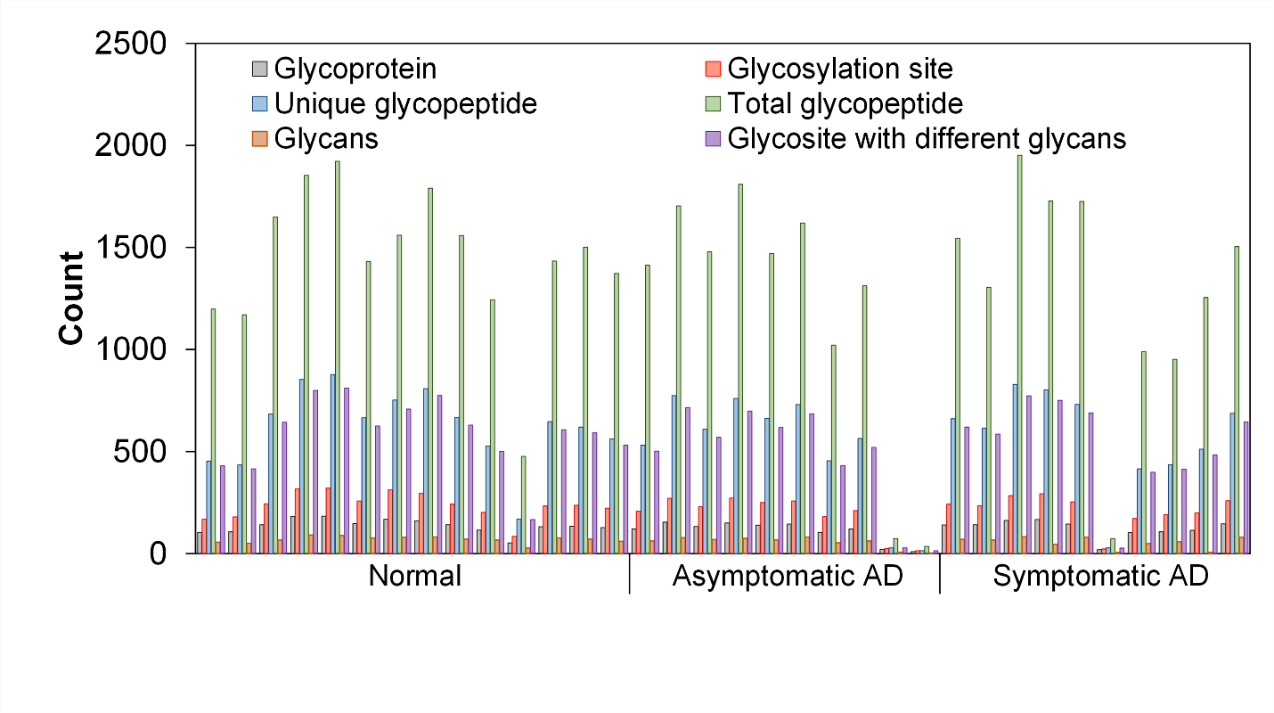
**

**Figure S5. Identification of glycospecies from thirty human brains.** Bar plot shows the number of glycoproteins, glycopeptides, glycans, glycosylation sites, and glycoforms detected in the 30 brain samples.

**
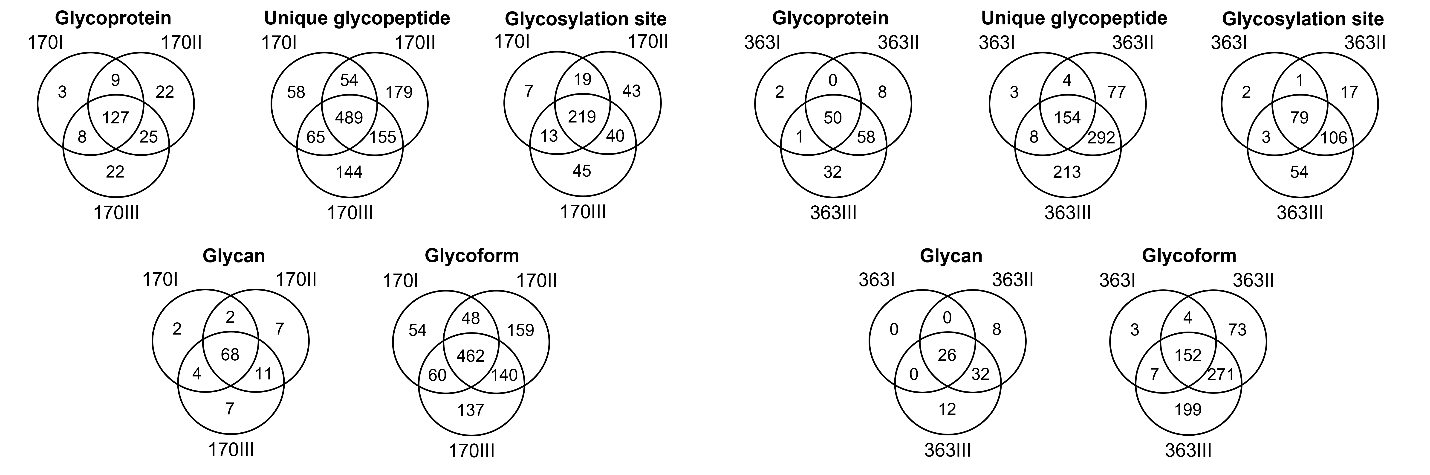
**

**Figure S6. Overlap of glycospecies from technical replicates.** Glycospecies identified from three technical runs of samples 170 and 363. The three replicates are shown as sample number I, II, or III in the figure and table.


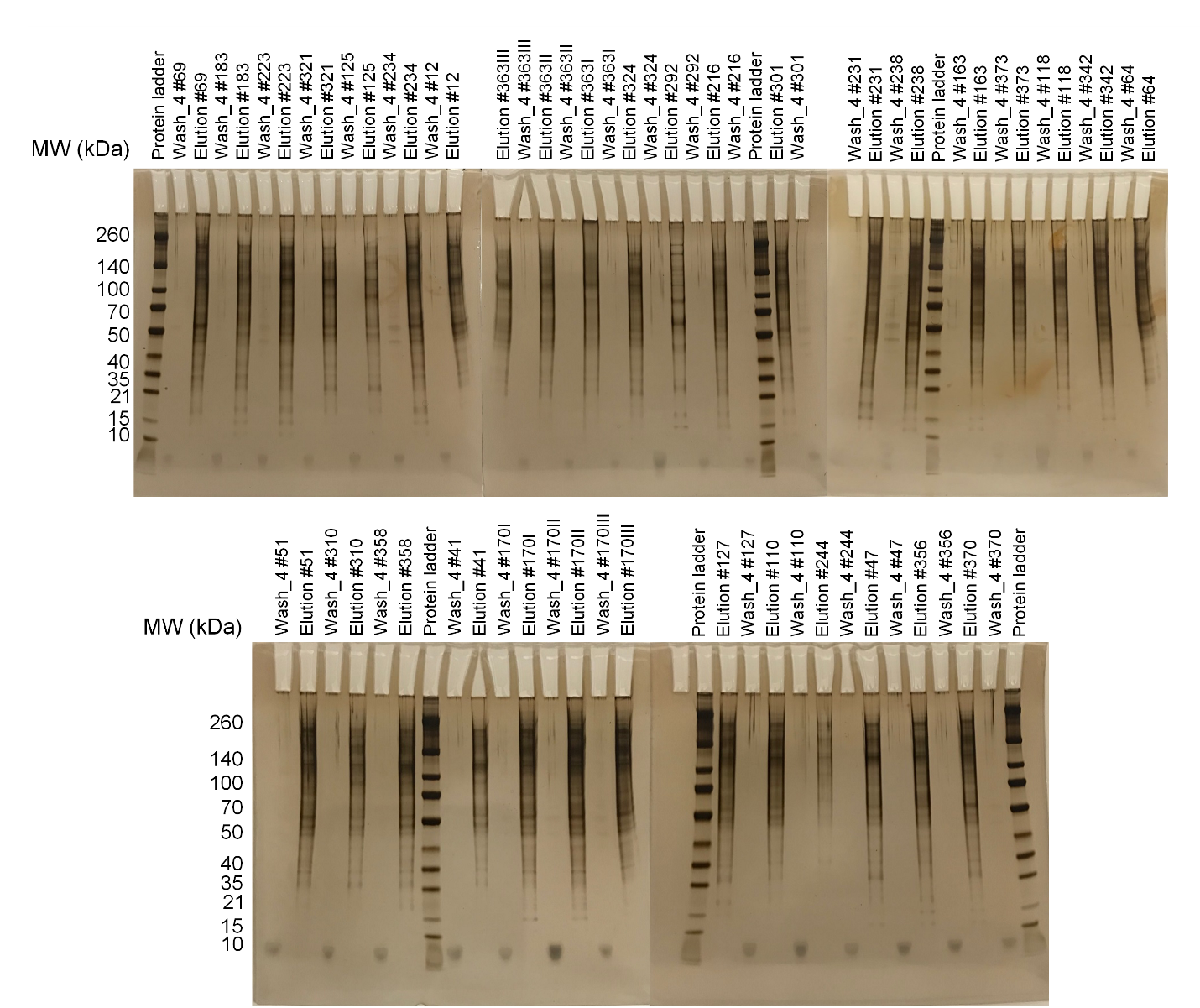


**Figure S7.** **Enrichment of glycoproteins from human brain samples.** Silver-stained SDS-PAGE of proteins in the wash or eluates from the 30 brain samples.


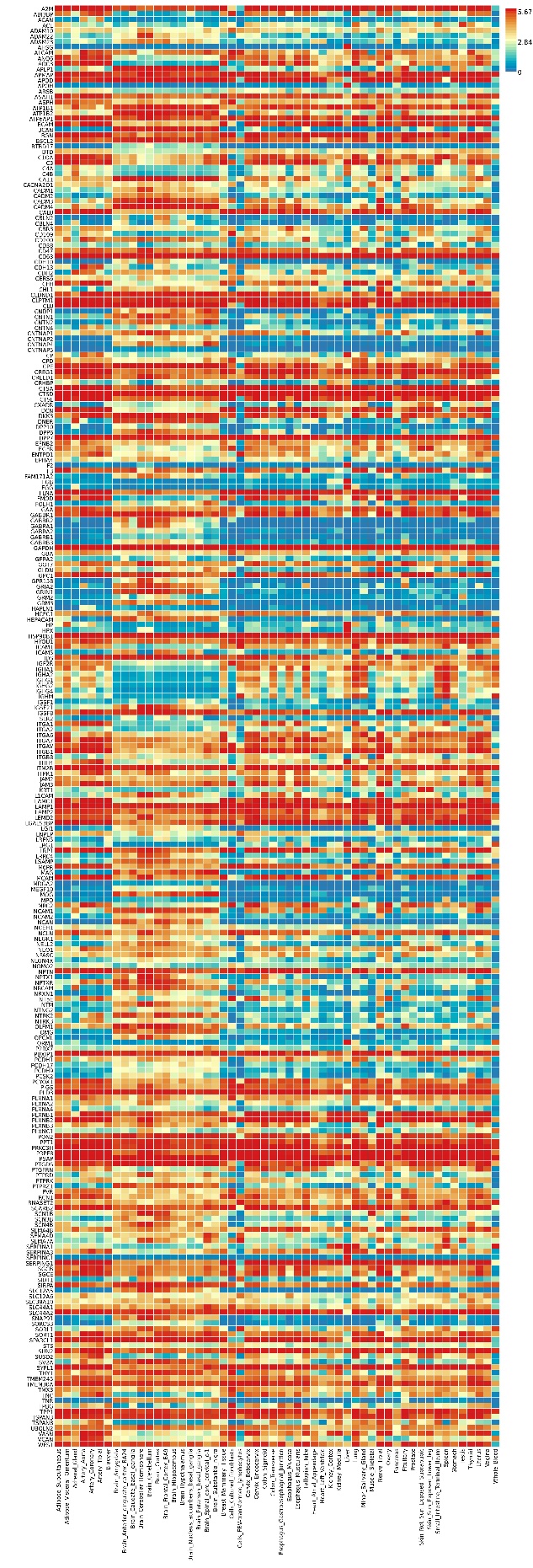


**Figure S8. Tissue expression of glycoproteins.** Heatmap showing tissue expression of glycoproteins based on FUMA GTEx V8 analysis against 54 tissues.

**
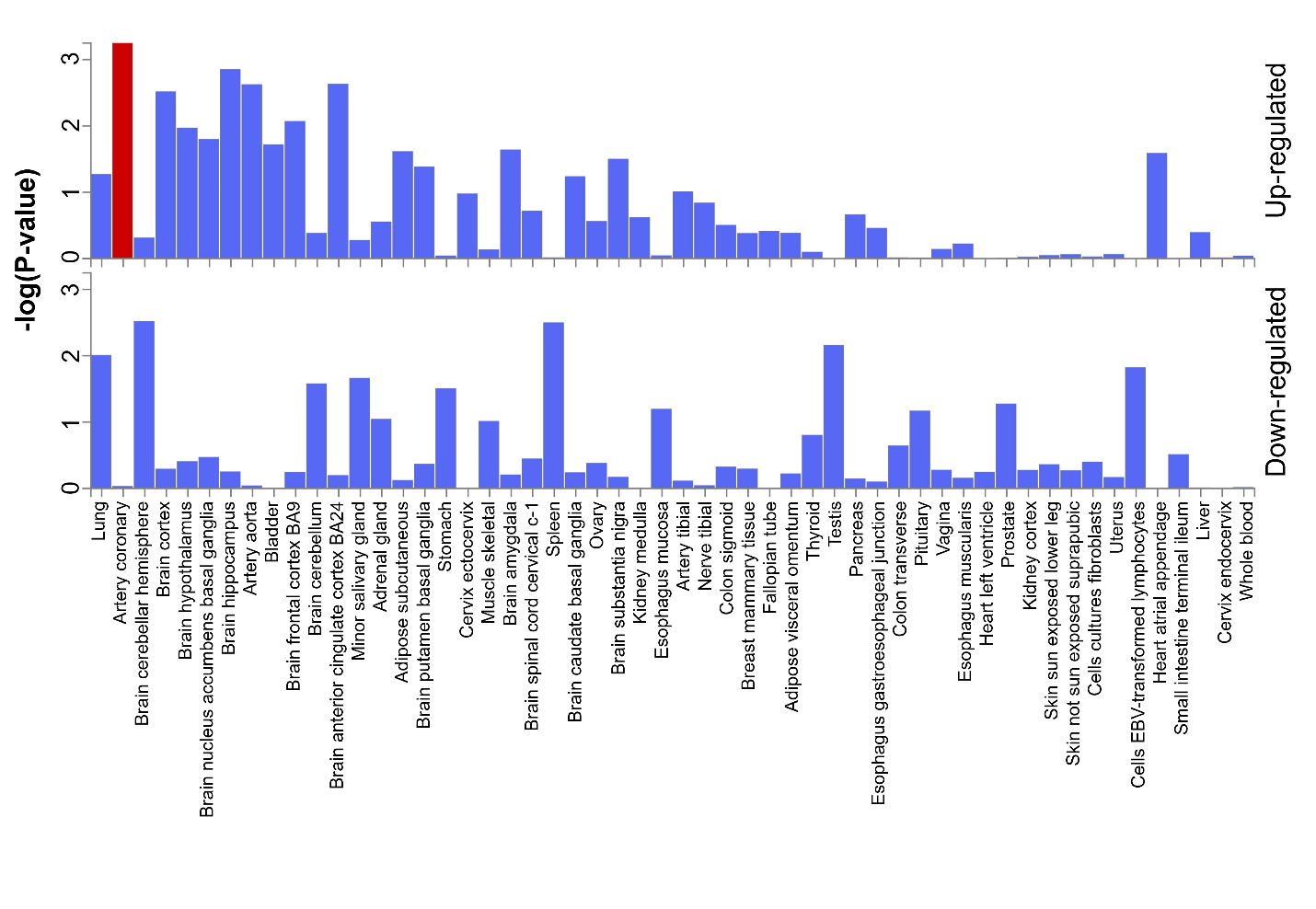
**

**Figure S9. Tissue expression of glycoproteins.** Tissue expression of glycoproteins identified in specific tissue types based on FUMA GTEx V8 analysis against 54 tissues. The red bar shows the significant tissue enrichment.


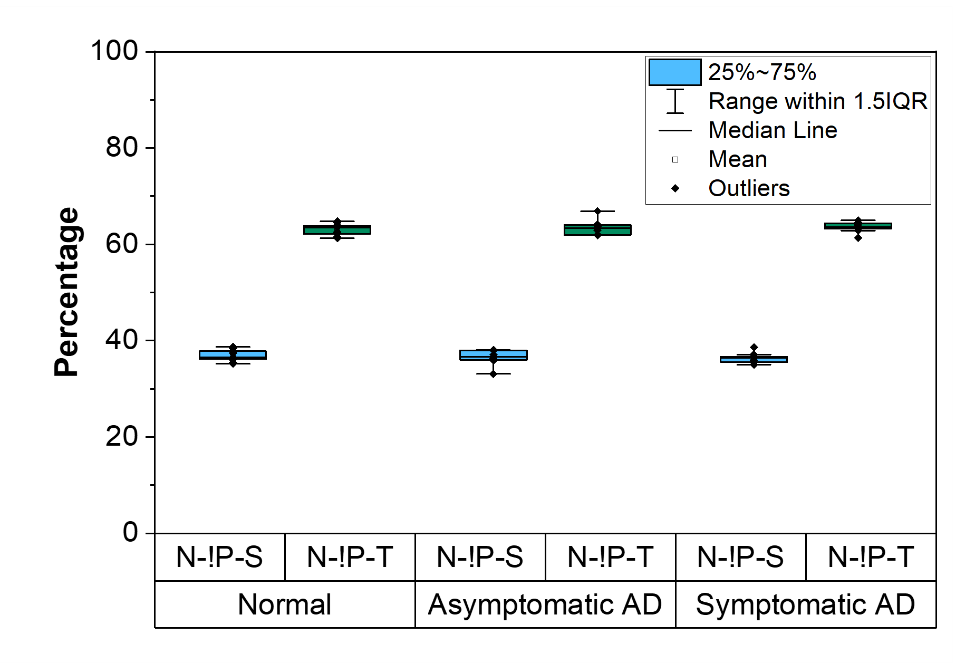


**Figure S10.** **Consensus motifs for N-linked glycosylation.** Distribution of N-glycosylation sites pooled from normal, asymptomatic, or symptomatic AD brains with consensus sequences N-!P-S/T. !P shows that the amino acid can be anything except proline.

**
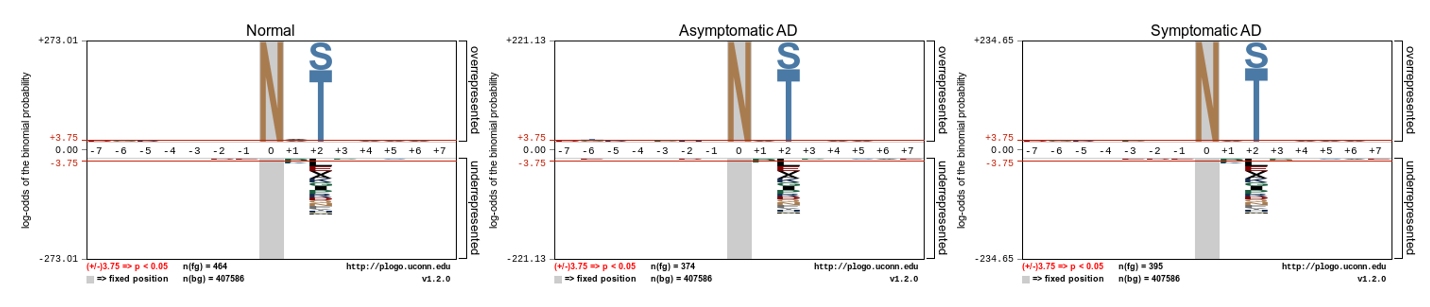
**

**Figure S11. Glycosylation sequence logo.** Sequence motifs enriched from the identified glycosylation sites from the three sample types. Sequence logos were generated with pLogo.

**
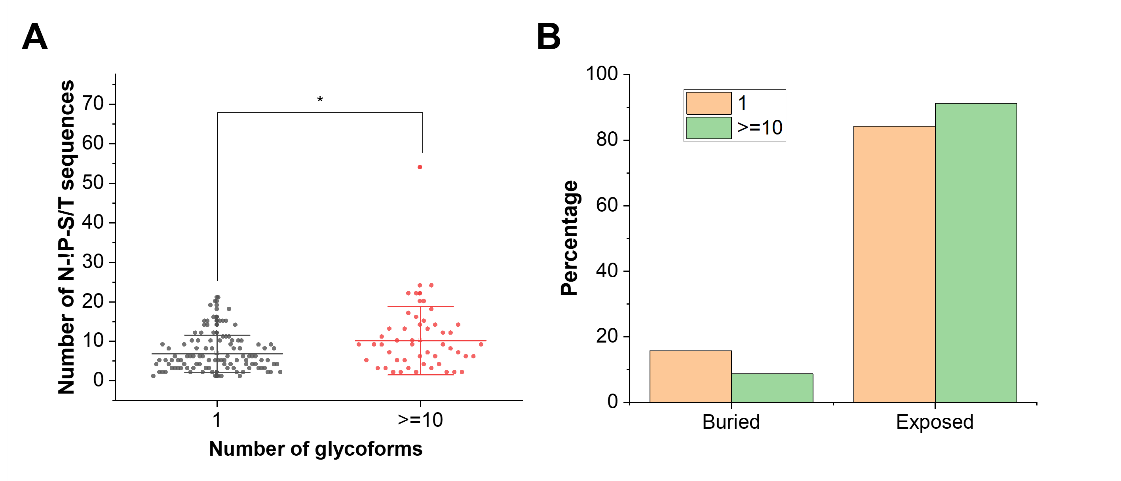
**

**Figure S12.** **Comparison of proteins with one or ten or more glycoforms.** (A) Comparison of N-!P-S/T sequences. The number of N-!P-S/T sequences from proteins with one or with ten or more glycoforms. The asterisk indicates P=0.0024464 by Student’s t-test. (B) Percentage of glycosylation sites predicted by NetSurf P to be exposed from the solvent or buried within proteins.

**
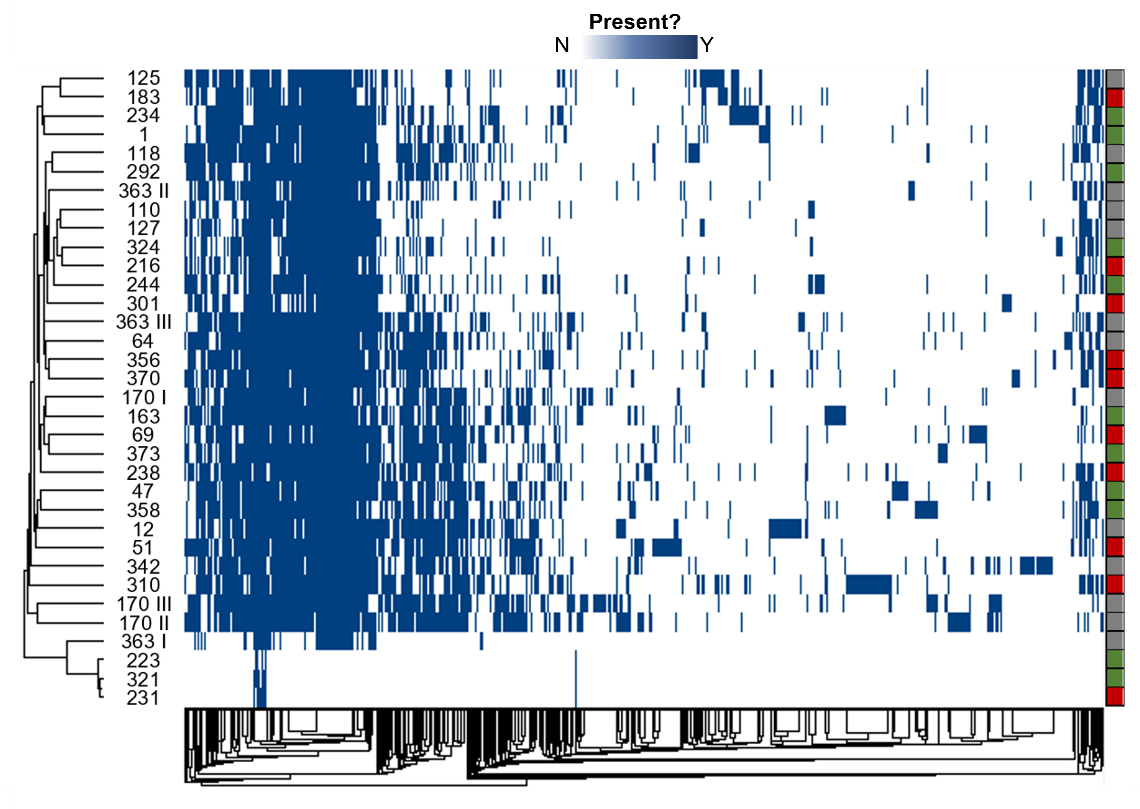
**

**Figure S13. N-linked glycosylation in individual human brains.** Heat map with unsupervised hierarchical clustering of glycosylation on glycoproteins detected from human brains. Each column represents UniProt ID + glycosylation site + glycan composition, such as O14594 + N122 + HexNAc(2)Hex(5). The color-coded column shows the corresponding sample type including normal (grey), asymptomatic AD (green), and symptomatic AD (red).


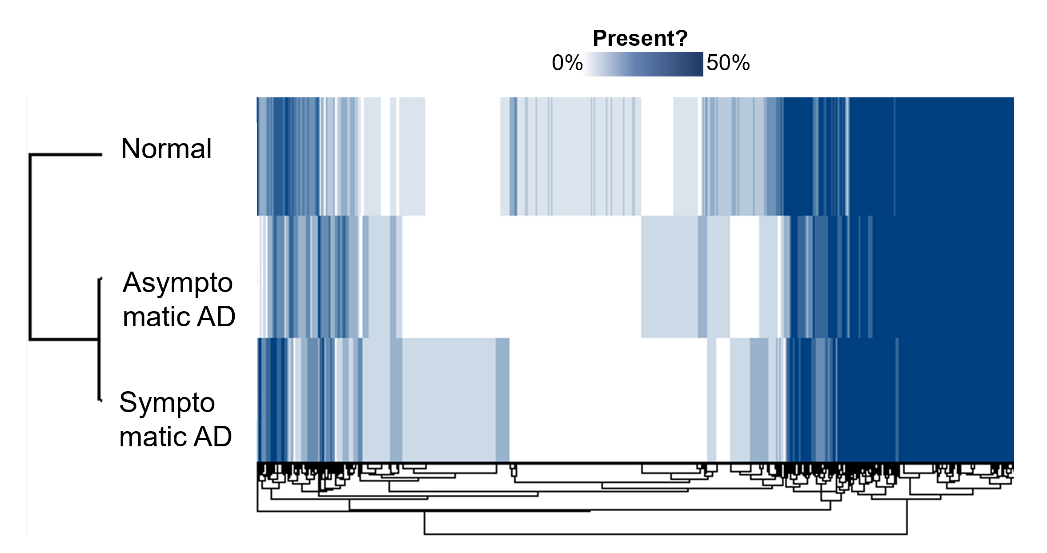


**Figure S14. N-linked glycosylation in human brains from each brain type.** Heat map with unsupervised hierarchical clustering of glycosylation on glycoproteins detected from each sample type, i.e., normal, asymptomatic AD, or symptomatic AD similar to the previous figure. Each column represents UniProt ID + glycosylation site + glycan composition, such as O14594 + N122 + HexNAc(2)Hex(5). Here, glycosylation at a particular glycosylation site for each sample type was pooled and compared.

**
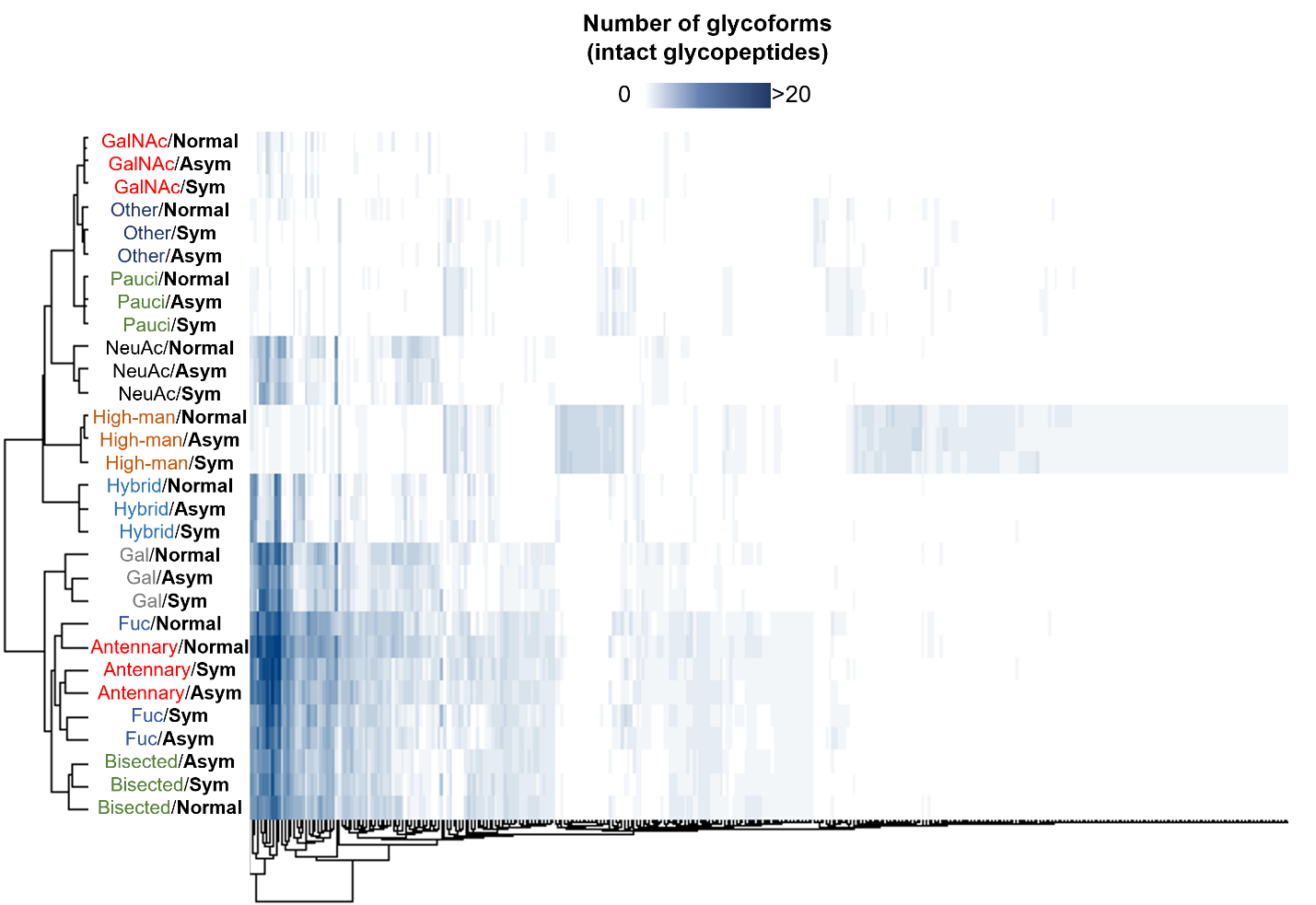
**

**Figure S15.** **N-linked glycan types in human brains.** Heat map with hierarchical clustering of glycan types presented on glycosylation sites. Each column shows a specific glycosylation site, e.g., O14594 + N122 and the number of glycans for that particular feature.


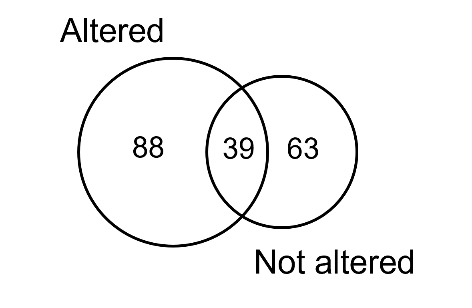


**Figure S16. Overlap of proteins with/with no altered glycosylation across the normal, asymptomatic, and symptomatic AD samples.** There is some overlap due to some glycosylation sites that may/may not have the same glycosylation profile.


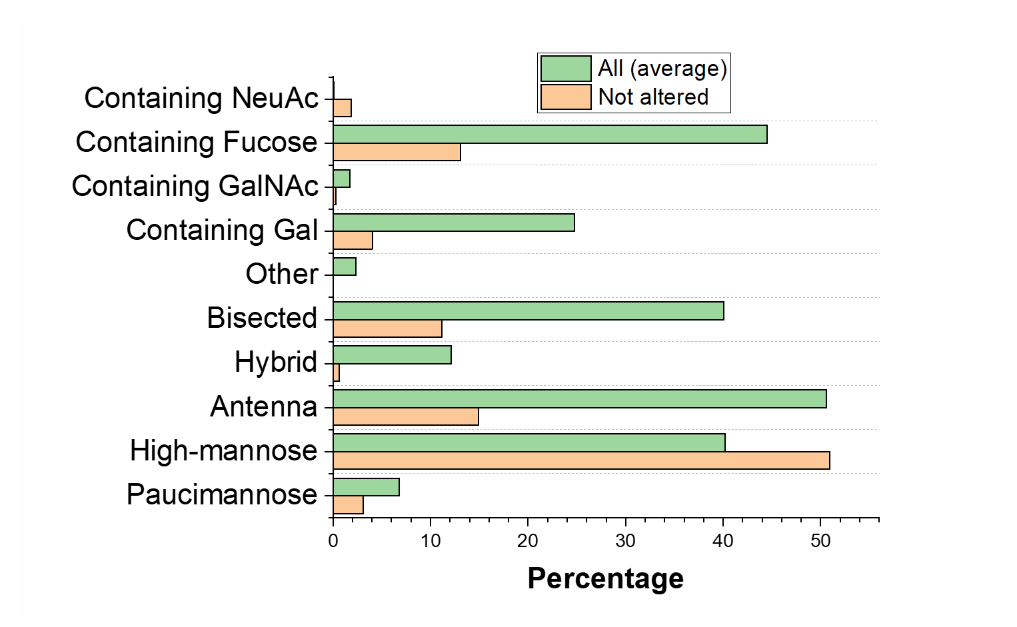


**Figure S17. Glycans on glycoproteins with no alteration in protein glycosylation.** Glycan types on one-third of the glycosylation sites from the heat map without changes in glycosylation were compared all glycans from all samples (averaged from **Figure 4A**).


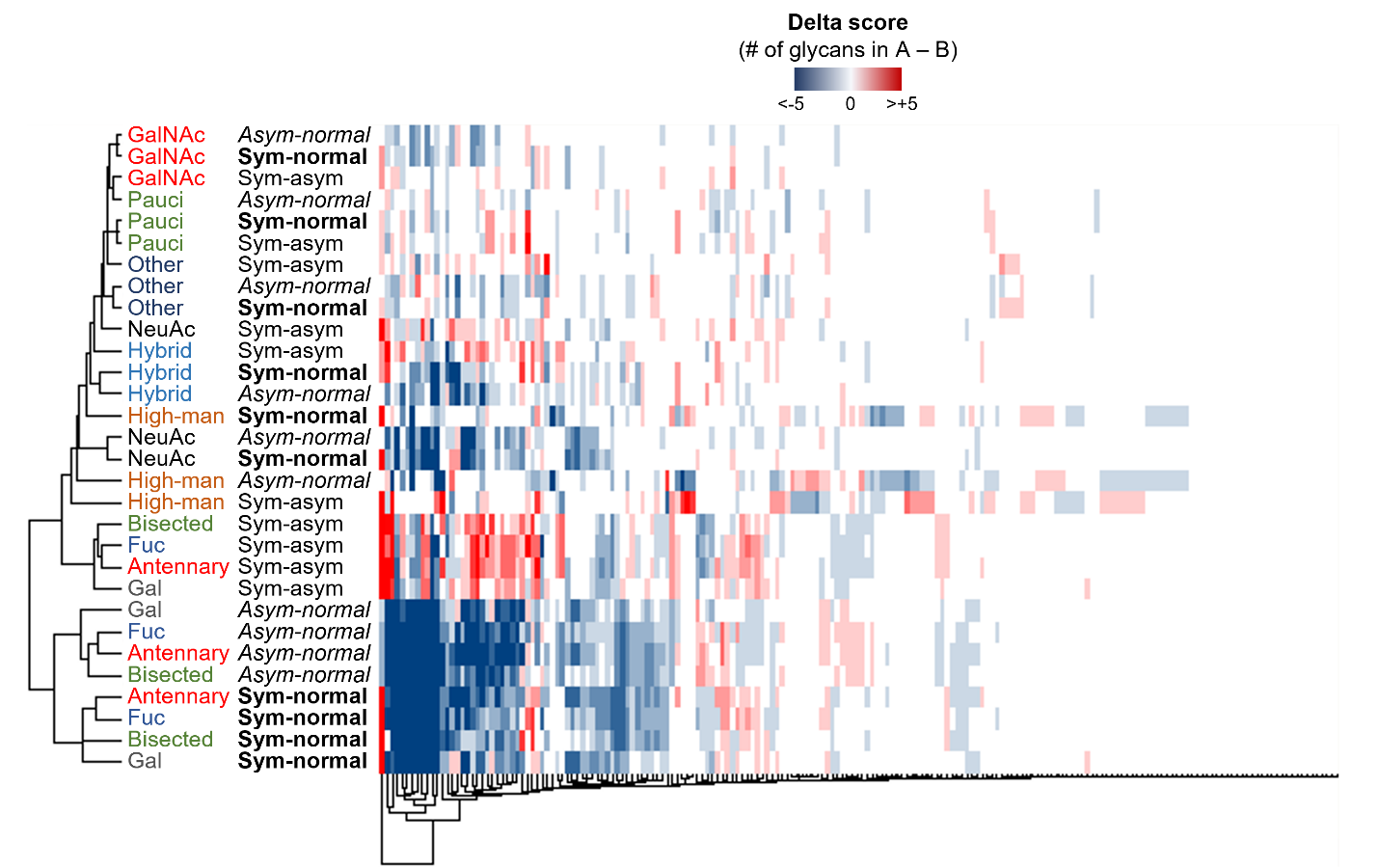


**Figure S18. Heat map of glycosylation alteration at the protein level.** Heat map with hierarchical clustering of glycan types presented on glycoproteins. Each column shows a specific glycoprotein and the difference in glycosylation features for any sample pairs. The delta score refers to the number of glycans from the first sample type – the number of glycans in the second sample type.


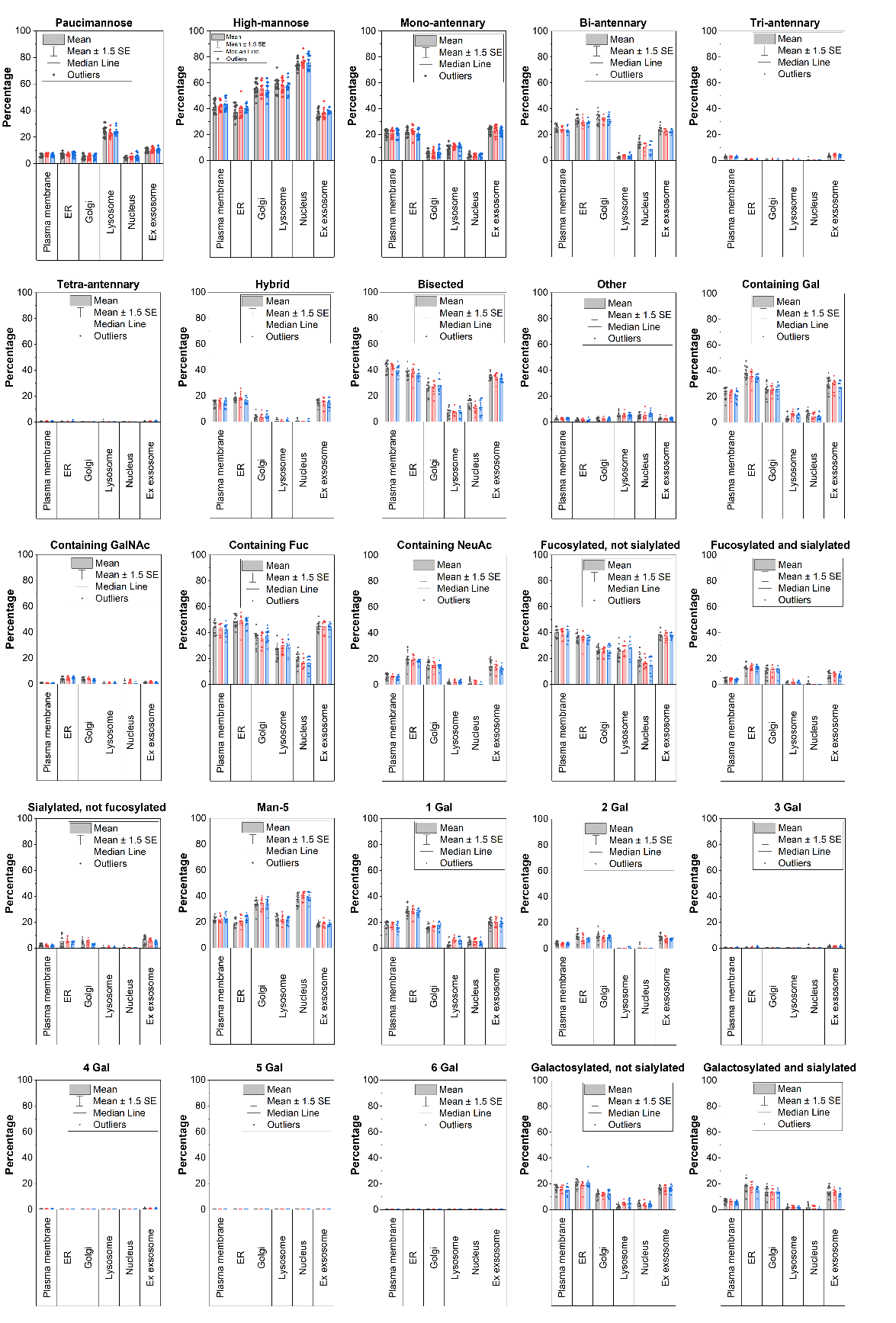


**Figure S19. Distribution of glycan types (glycoforms) among the cellular compartments.** The comparison shown here is similar to **Figure 5** but the same glycan types are compared here.

**
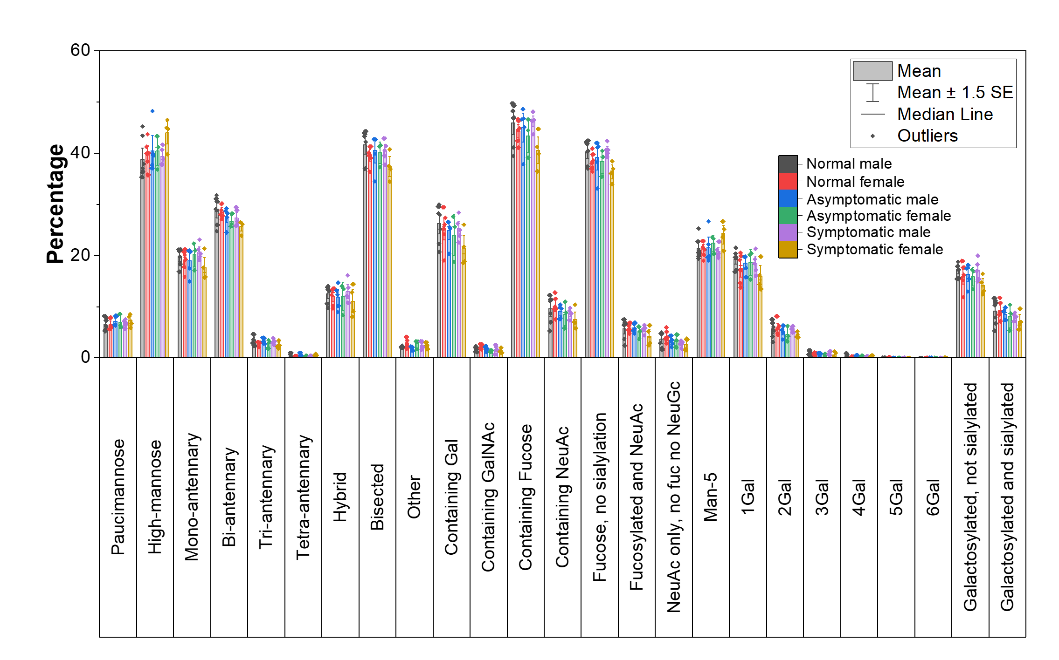
**

**Figure S20. Distribution of glycoforms in samples separated by sexes.** The distribution of glycan types in male or female normal, asymptomatic, and asymptomatic AD samples is shown. ANOVA shows no difference in glycan type distribution for each glycan type.


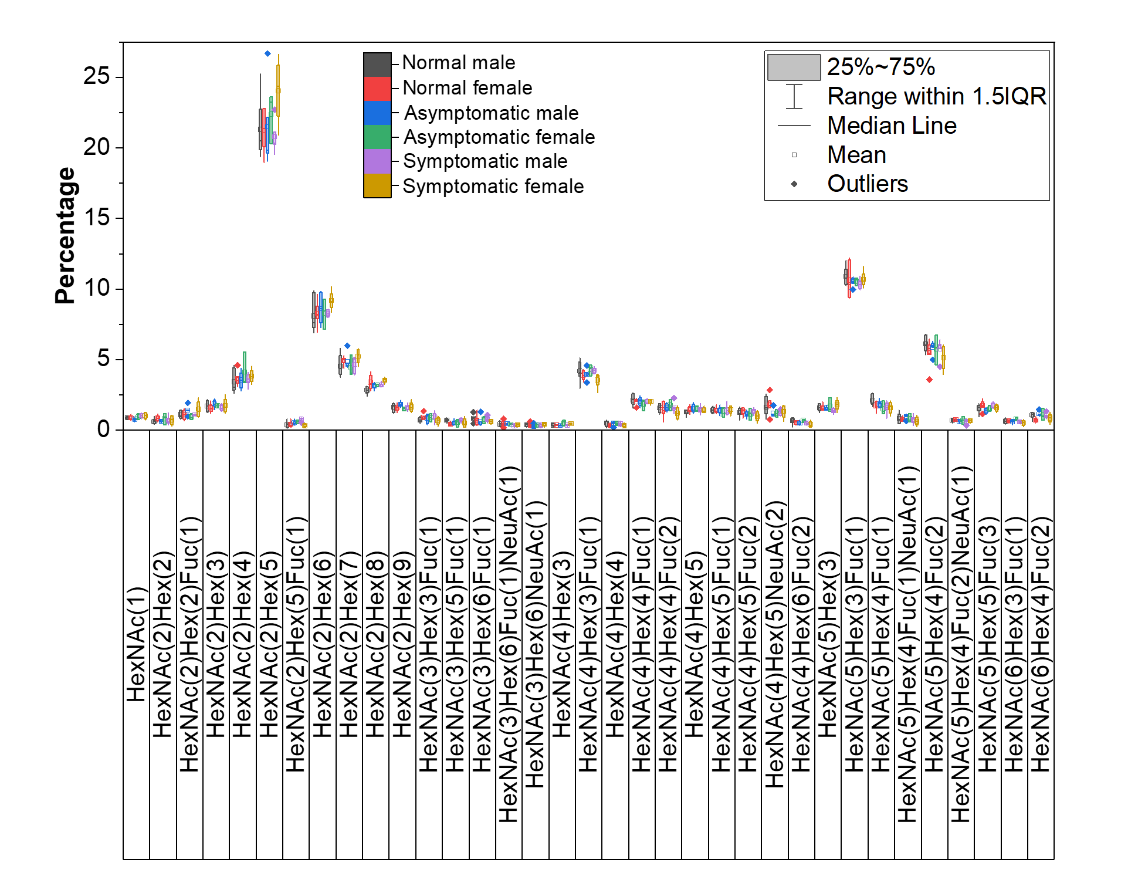


**Figure S21. Distribution of commonly identified glycans in samples separated by sexes.** The distribution of glycans in male or female normal, asymptomatic, and asymptomatic AD samples is shown. ANOVA shows no difference in glycan type distribution for each glycan.


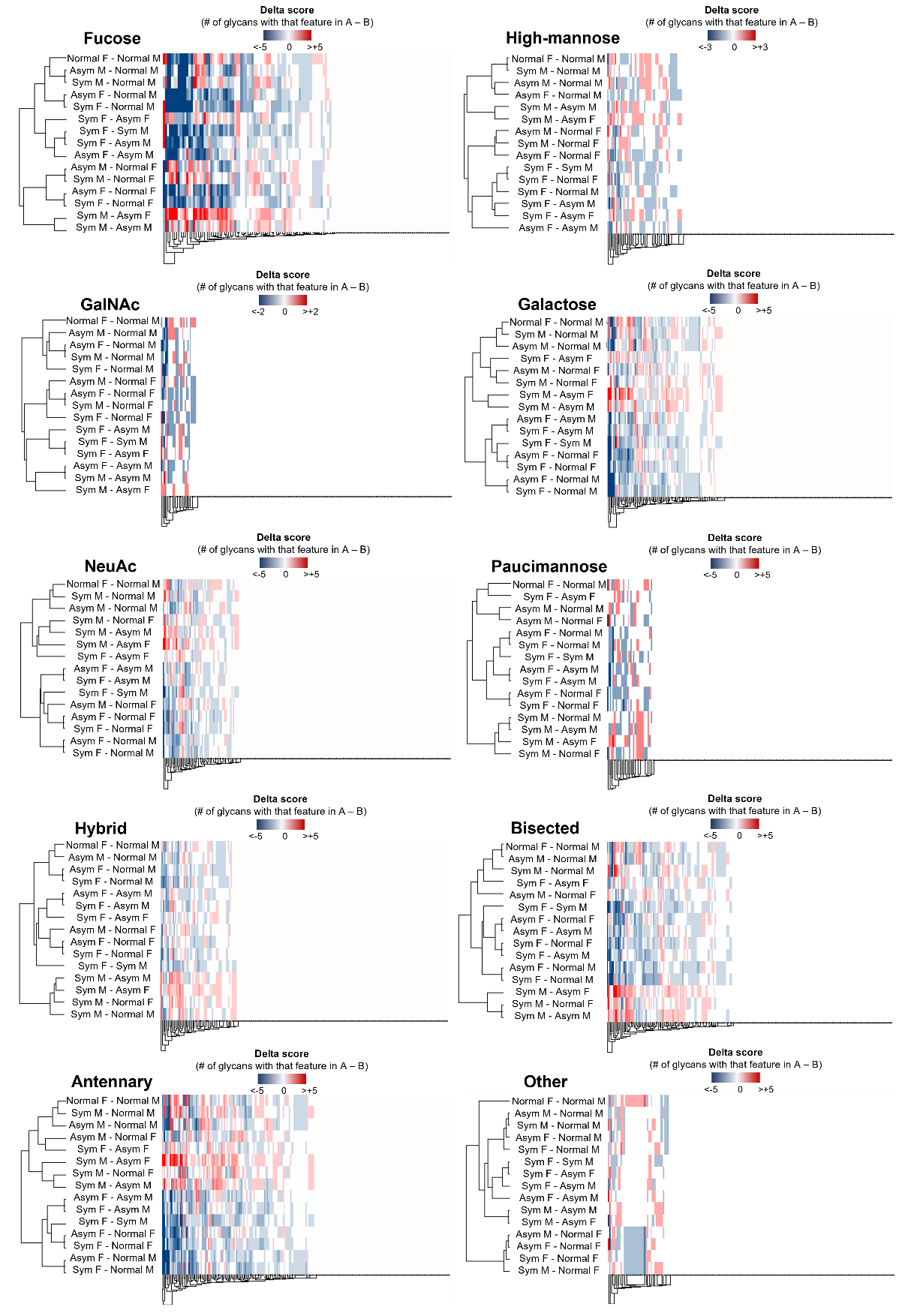


**Figure S22. Heat map of delta score for each glycan type from samples separated by sex.** Heat map with hierarchical clustering of glycan types presented on glycosylation sites. Each column shows a specific glycosylation site.

**
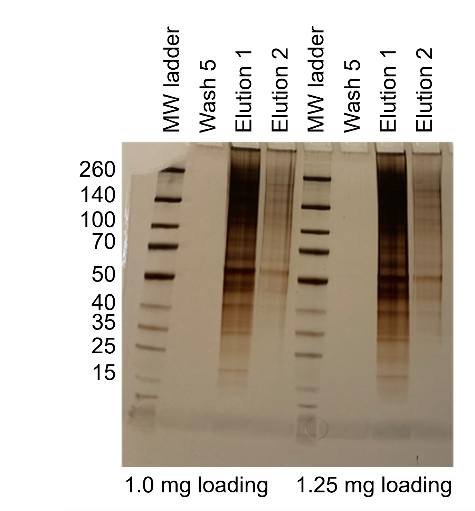
**

**Figure S23.** **Enrichment of O-GlcNAcylated glycoproteins from human brains.** Silver-stained SDS-PAGE of WGA-enriched proteins for O-GlcNAcylated peptide analysis with different starting protein amounts. The wash and eluates were separate to show the enrichment efficiency.
